# Cellular determinants influence the red blood cell adsorption efficiency of poly(amine-*co*-ester) nanoparticles

**DOI:** 10.1126/sciadv.adt8637

**Published:** 2025-05-02

**Authors:** Thomas C. Binns, David A. Eaton, Dana V. Akiki, Emily Deschenes, Alexandra S. Piotrowski-Daspit, Laura G. Bracaglia, Jeanne E. Hendrickson, W. Mark Saltzman

**Affiliations:** ^1^ Department of Laboratory Medicine, Yale University, New Haven, CT 06520, USA.; ^2^ Department of Biomedical Engineering, Yale University, New Haven, CT 06520, USA.; ^3^ Department of Anesthesiology, Perioperative Care, and Pain Medicine, NYU Grossman School of Medicine, New York, NY 10016, USA.; ^4^ Department of Biomedical Engineering, University of Michigan, Ann Arbor, MI 48109, USA.; ^5^ Department of Chemical and Biological Engineering, Villanova University, Villanova, PA 19085, USA.; ^6^ Department of Pathology and Laboratory Medicine, Emory University, Atlanta, GA 30322, USA.; ^7^ Department of Chemical & Environmental Engineering, Yale University, New Haven, CT 06520, USA.; ^8^ Department of Cellular & Molecular Physiology, Yale University, New Haven, CT 06520, USA.

## Abstract

Many poly(amine-*co*-ester) (PACE) nanoparticles, drug delivery vehicles for nucleic acid and small molecule cargoes, accumulate in the liver and spleen following intravenous administration, limiting delivery to nonhepatosplenic tissues. Red blood cell (RBC) hitchhiking, a strategy in which nanoparticles are nonspecifically adsorbed to RBCs prior to administration, has been used to modulate nanoparticle biodistribution, enabling enrichment in organs immediately downstream from the site of vascular infusion. We find that scarcely investigated cellular determinants—namely, storage duration, membrane stiffness, and membrane-bound sialic acid quantity—substantially affect PACE nanoparticle adsorption efficiency. Following development of an optimized adsorption protocol, RBC hitchhiking was shown to enhance PACE nanoparticle cargo delivery to pulmonary tissue while also increasing exposure to other assayed organs. These findings inform future RBC hitchhiking study design, implicate cellular variables as potential obstacles or boons to clinical translation, and demonstrate the delivery of nucleic acids using this strategy with the PACE nanoparticle platform.

## Teaser

Erythrocyte properties influence the cell-mediated delivery of drug-loaded nanoparticles intended for nonhepatic enrichment.

## INTRODUCTION

Nanoparticle drug delivery systems enable the modulation of drug pharmacokinetics and biodistribution (PK/BD) by decoupling a drug’s fate from its physiochemical properties and associating it with those of the nanoparticle. Polymeric nanoparticles can encapsulate small molecule, protein, or nucleic acid cargoes and provide a wide chemical design space for tuning PK/BD. However, unless specifically engineered to avoid uptake by the mononuclear phagocyte system, intravenously administered polymeric nanoparticles generally accumulate in the liver and spleen (*1*). Strategies to minimize hepatosplenic uptake include conjugating active-targeting moieties (e.g., antibodies) to the surface of nanoparticles, administering “decoy” materials to saturate phagocytes prior to nanoparticle administration, or depleting phagocytes prior to administration—an approach confined to experimental animal use (*1*).

Cell-facilitated drug delivery also provides a mechanism for PK/BD modulation and nonhepatosplenic enrichment (*2*–*4*). One strategy reported in a growing number of publications uses red blood cells (RBCs) as carriers for nonspecifically adsorbed nanoparticles—a method commonly referred to as “RBC hitchhiking” (*2*–*27*). This strategy has been used to modulate nanoparticle circulation half-life and biodistribution, promoting deposition in the microcapillary bed immediately downstream from the site of intravascular administration via a “first-pass” effect (*5*, *16*).

RBC hitchhiking has two major strengths: (i) the preexistence of infrastructure enabling the collection, processing, storage, and distribution of allogeneic and autologous RBCs, and (ii) decades of transfusion medicine research that inform the challenges to and solutions for implementing clinical translation of this strategy. A concept fundamental in the transfusion medicine literature is that of a “storage lesion”—the deleterious changes in cellular characteristics that occur during the storage of blood products. As storage duration increases, RBCs exhibit increased stiffness, increased spherocytic/echinocytic morphology, and decreased zeta potential magnitude (i.e., less negative), which correlate with increased hemolysis susceptibility and faster in vivo clearance (*28*–*33*).

Although prior reports have described investigations into how nanoparticle properties influence RBC adsorption efficiency (*5*, *7*–*9*, *11*–*13*, *16*–*20*, *25*), few have investigated how cellular characteristics influence polymeric nanoparticle-RBC adsorption, specifically those associated with the RBC storage lesion (*9*, *20*, *25*). These cellular determinants are important variables to consider in RBC hitchhiking studies and will be important when developing manufacturing protocols for clinical translation using either stored allogeneic donor RBCs or autologously collected RBCs.

Polymeric nanoparticles with demonstrated RBC hitchhiking compatibility include those made from polystyrene, poly(lactic-*co*-glycolic) acid (PLGA), polyethylenimine (PEI), and chitosan (*5*–*7*, *9*, *14*, *16*–*20*, *23*–*26*). These systems have been used to deliver small molecule and protein therapeutics for the treatment of several animal models of disease (*16*–*18*, *20*, *23*, *24*, *26*). Nucleic acid cargoes have yet to be investigated in conjunction with the RBC hitchhiking strategy. In this study, we report the compatibility of a subset of poly(amine-*co*-ester) (PACE) nanoparticles, mildly cationic particles with a demonstrated capacity to deliver therapeutic nucleic acids efficiently, with the RBC hitchhiking strategy (*1*, *34*–*45*). We describe how the RBC storage lesion and associated cellular determinants correlate with PACE nanoparticle-RBC adsorption efficiency. These findings have implications for the design of future RBC hitchhiking studies and the clinical translation of this approach. Last, we demonstrate small molecule and nucleic acid cargo enrichment in visceral organs, particularly the lungs, following intravenous administration of RBC-adsorbed PACE nanoparticles as compared to free nanoparticles.

## RESULTS

### PACE nanoparticles can be adsorbed onto RBCs

Unless otherwise noted, the primary “PACE60” nanoparticle formulation used in this study consisted of PACE polymer synthesized with a 60 mol % feed fraction of 15-pentadecanolide [PDL; (*38*)] and encapsulated Cy5-tagged single-stranded DNA (ssDNA) resulting in particles ranging from ~200 to 300 nm in diameter (table S1), consistent with previously reported morphology (*38*, *39*). The standard RBC adsorption protocol is detailed in the Materials and Methods section. Briefly, nanoparticles were mixed with washed murine C57BL/6 RBCs at a ratio of 100:1 in Dulbecco’s phosphate-buffered saline (DPBS, -Ca, -Mg) and incubated for 1 hour at 4°C, gently mixing every 15 min. RBC-nanoparticle complexes were subsequently subjected to three high-volume (~10x) chilled DPBS washes to remove unbound particles.

**Fig. 1.**
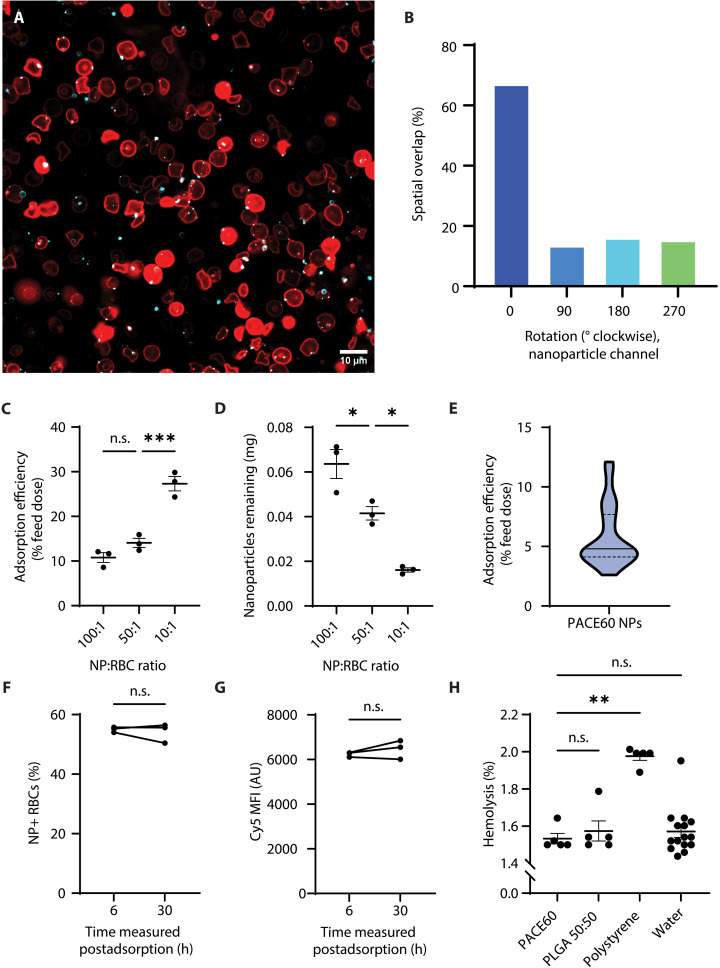
PACE60 nanoparticle-RBC adsorption characterization. (**A**) Representative confocal micrograph of PACE60 nanoparticles adsorbed to the surface of RBCs; scale bar = 10 μm. (**B**) Percentage of nanoparticle channel signal overlapping with RBC channel signal as a function of RBC channel 90° clockwise image rotation (internal colocalization controls); *n* = 1. (**C**) Adsorption efficiency of PACE60 nanoparticles as a function of NP:RBC feed ratio; *n* = 3. (**D**) Nanoparticle mass adsorbed as a function of NP:RBC feed ratio; *n* = 3. (**E**) Compiled adsorption efficiency data of PACE60 nanoparticles adsorbed via the standard protocol throughout this report. Solid line: median; dashed lines: interquartile range; *n* = 29. (**F**) Percentage of RBCs with associated nanoparticle signal as a function of time between adsorption completion and flow cytometric measurement; *n* = 3. (**G**) Median nanoparticle fluorescence intensity of nanoparticle-associated RBCs as a function of time between adsorption completion and flow cytometric measurement; *n* = 3. (**H**) Adsorption induced hemolysis as a function of nanoparticle composition or nanoparticle suspension vehicle (water); *n* = 5 to 15. *P < 0.05; **P < 0.01; ***P < 0.001, n.s., not significant. Lines and error bars in individual data point plots indicate means ± SEM. See Materials and Methods for micrograph processing methodology. NP, nanoparticle; RBC, red blood cell; PACE60, poly(amine-*co*-ester) synthesized with 60 mol % PDL; MFI, median fluorescence intensity; AU, arbitrary units; PLGA 50:50, poly(lactic-*co*-glycolic) acid synthesized with a 1:1 monomer ratio. h, hours.

Confocal imaging of PACE60 nanoparticles mixed as above with DiO-, membrane-labeled RBCs revealed nanoparticles associated with RBC membranes (Fig. 1A). A video montage displaying a *z*-stack animation of several RBCs isolated from this field of view (FOV) is available in the Supplementary Materials (movie S1). No substantial rouleaux or RBC aggregation was noted in imaged specimens. Association was confirmed visually and quantitatively: 67.0% of the nanoparticle signal was shown to overlap with RBC membrane signal, as compared to 13.4, 16.0, and 15.2% when the imaged RBC channel was rotated 90°, 180°, and 270° clockwise as internal colocalization controls, respectively (Fig. 1B and fig. S1) (*46*). A quantitative pipeline enabling the measurement of adsorption efficiency (i.e., percent nanoparticle feed signal remaining following postadsorption washes), percent RBCs with associated nanoparticles, and relative nanoparticle signal per nanoparticle-positive RBC was developed. PACE60 nanoparticle adsorption efficiency was found to be inversely correlated with nanoparticle-to-RBC (NP:RBC) ratio (Fig. 1C). The increase in efficiency observed at lower NP:RBC ratios did not, however, overcome the reduced absolute mass of adsorbed nanoparticles observed at lower NP:RBC ratios (Fig. 1D).

Compiling all standard protocol (NP:RBC = 100:1) adsorption efficiency measures reported in this study revealed a positively skewed distribution with a median (interquartile range) adsorption efficiency of 4.8% (4.1 to 7.7%) (Fig. 1E). As measured by flow cytometry 6 hours following adsorption, ~55% of RBCs were associated with nanoparticles (Fig. 1F). This interaction was robust over time. No significant differences were detected in the proportion of nanoparticle-positive RBCs nor the Cy5 median fluorescence intensity (MFI) of RBC-nanoparticle complexes stored at 4°C for 30 hours postadsorption (Fig. 1, F and G). Adsorption was also robust with respect to shear forces generated by washing. Most nonadhered particles were removed following solution centrifugation, supernatant removal, and one subsequent DPBS wash. Subsequent washes did not remove a significant portion of nanoparticles (fig. S2).

Minimal hemolysis was noted following adsorption of PACE60 nanoparticles. The amount of hemolysis measured as a result of adsorption did not differ significantly between PACE60 and PLGA nanoparticles nor the water (nanoparticle stock suspension medium) control. Water volume added to RBC suspensions was minimized, with relative tonicity changes limited to ≤ 5%. Adsorption of PACE60 nanoparticles induced less hemolysis than adsorption of polystyrene particles (Fig. 1H).

### PACE60 nanoparticle adsorption efficiency is inversely correlated with RBC storage duration

**Fig. 2.**
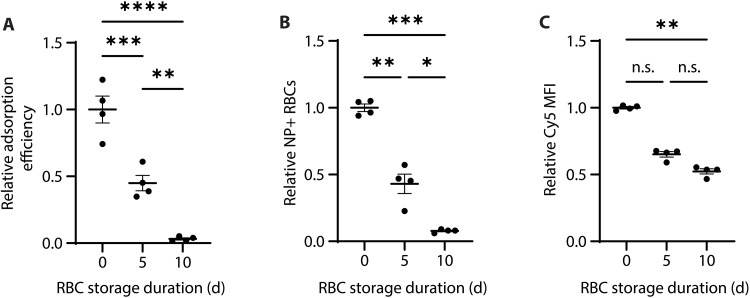
PACE60 nanoparticle-RBC adsorption characterization as a function of RBC storage duration. (**A**) Relative adsorption efficiency of PACE60 nanoparticles as a function of RBC storage duration. (**B**) Relative percentage of RBCs with associated nanoparticle signal as a function of RBC storage duration. (**C**) Relative median nanoparticle fluorescence intensity of nanoparticle-associated RBCs as a function of RBC storage duration. *n* = 4; *P < 0.05; **P < 0.01; ***P < 0.001; ****P < 0.0001; n.s., not significant. Lines and error bars in individual data point plots indicate means ± SEM. PACE60, poly(amine-*co*-ester) synthesized with 60 mol % PDL; RBC, red blood cell; NP, nanoparticle; MFI, median fluorescence intensity; d, days.

Using stored RBCs as an adsorption substrate—as compared to freshly collected RBCs—drastically decreased nanoparticle adsorption efficiency. After 5 days of storage, nanoparticle adsorption efficiency decreased by an average of 55% compared to adsorption onto fresh RBCs. Efficiency dropped by 97% following adsorption to RBCs stored for 10 days (Fig. 2A). This trend was also observed at similar magnitudes in flow cytometric measures of the proportion of RBCs with associated nanoparticles (Fig. 2B). This proportion decreased by 57 and 92% in 5- and 10-day-stored RBC groups, respectively, as compared to the fresh RBC group. Measures of the Cy5 MFI of nanoparticle-associated RBCs showed the same trend at a reduced magnitude. The average Cy5 MFI of nanoparticle-associated RBCs from the 5-day-old RBC group was 65% that of the fresh RBC group (*P* = 0.3481, not significant), whereas that of the 10-day-old RBC group was 52% (*P* = 0.0050; Fig. 2C). Together, these findings suggest that using stored RBCs as compared to fresh RBCs resulted in a smaller proportion of RBCs with associated nanoparticles and fewer nanoparticles present on RBC-nanoparticle complexes.

### RBC stiffness is variably correlated with PACE60 nanoparticle adsorption efficiency

**Fig. 3.**
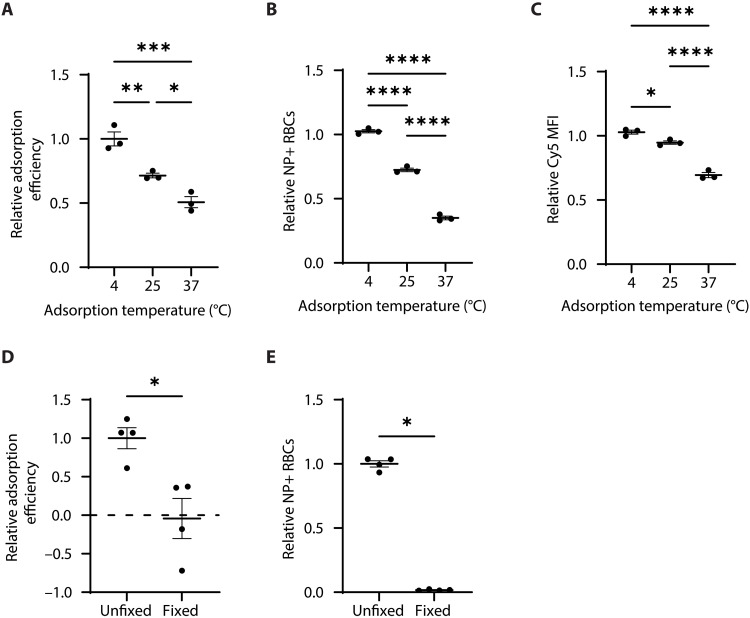
PACE60 nanoparticle-RBC adsorption characterization as a function of membrane stiffness modulation. (**A**) Relative adsorption efficiency of PACE60 nanoparticles as a function of adsorption temperature. (**B**) Relative percentage of RBCs with associated nanoparticle signal as a function of adsorption temperature. (**C**) Relative median nanoparticle fluorescence intensity of nanoparticle-associated RBCs as a function of adsorption temperature. (**D**) Relative adsorption efficiency of PACE60 nanoparticles as a function of RBC fixation. (**E**) Relative percentage of RBCs with associated nanoparticle signal as a function of RBC fixation. *n* = 3 for temperature experiments, *n* = 4 for fixation experiments; *P < 0.05; **P < 0.01; ***P < 0.001; ****P < 0.0001; n.s., not significant. Lines and error bars in individual data point plots indicate means ± SEM. PACE60, poly(amine-*co*-ester) synthesized with 60 mol % PDL; NP, nanoparticle; RBC, red blood cell; MFI, median fluorescence intensity.

RBC stiffness is associated with the storage lesion, so we sought to investigate its effects upon nanoparticle adsorption. RBC membrane stiffness, as measured by elastic moduli and the deformability index, increases with decreasing temperature (*47*–*49*). PACE60 nanoparticle adsorptions were carried out at 4°, 25°, or 37°C to observe the effect of temperature on adsorption efficiency. Adsorption efficiency decreased with increasing temperature as measured by fluorimetry and flow cytometry (Fig. 3, A to C). However, it is unlikely that changing the adsorption temperature exerts an isolated effect upon membrane stiffness. Other variables such as nanoparticle biophysical characteristics and entropy within the adsorption system influencing nanoparticle-RBC interaction/dissociation frequency may also be affected.

To avoid these various effects and modulate RBC stiffness in a more isolated manner, PACE60 nanoparticle adsorptions were carried out using glutaraldehyde-fixed RBCs. Glutaraldehyde fixation has been shown to drastically increase RBC stiffness, even beyond that induced by reducing temperature to 4°C (*50*). Fixation eliminated virtually all PACE60 nanoparticle adsorption capacity as measured by fluorimetry and flow cytometry (Fig. 3, D and E). Stiffness may not be the only variable modified by glutaraldehyde as fixation has been postulated to alter cellular zeta potential due to chemical alterations that facilitate protein cross-linking. However, we did not find any significant difference between fixed versus unfixed RBC zeta potential upon measurement by dynamic light scattering (DLS; fig. S3A).

### RBC membrane-bound sialic acid quantity is correlated with PACE60 nanoparticle adsorption efficiency

RBC membrane-bound sialic acid quantity is another variable associated with the storage lesion. Previous reports have demonstrated that, as RBC storage duration increases, zeta potential magnitude decreases, becoming more neutral (*30*, *51*, *52*). This reduction in zeta potential magnitude is driven by a reduction in membrane sialic acid content (*30*, *51*–*53*). We replicated these findings via DLS using murine RBCs stored for 0, 5, and 10 days (fig. S3B).

**Fig. 4.**
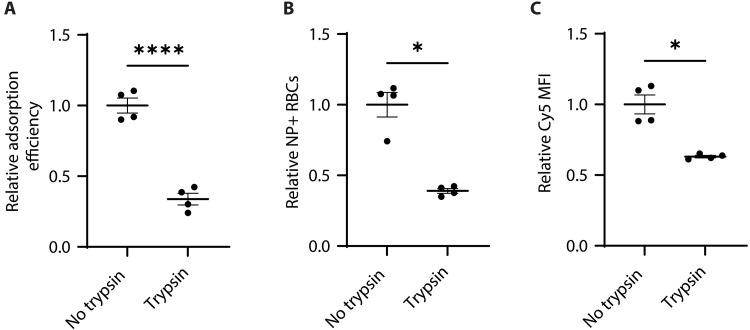
PACE60 nanoparticle-RBC adsorption characterization as a function of membrane sialoglycoprotein quantity modulation. (**A**) Relative adsorption efficiency of PACE60 nanoparticles as a function of RBC trypsin-EDTA treatment. (**B**) Relative percentage of RBCs with associated nanoparticle signal as a function of RBC trypsin-EDTA treatment. (**C**) Relative median nanoparticle fluorescence intensity of nanoparticle-associated RBCs as a function of RBC trypsin-EDTA treatment. *n* = 4; *P < 0.05; ****P < 0.0001. Lines and error bars in individual data point plots indicate means ± SEM. PACE60, poly(amine-*co*-ester) synthesized with 60 mol % PDL; NP, nanoparticle; RBC, red blood cell; MFI, median fluorescence intensity.

To investigate the effects of sialic acid quantity upon PACE60 nanoparticle-RBC adsorption efficiency, murine RBCs were treated with trypsin plus EDTA prior to adsorption and compared with nontreated RBC adsorptions. Trypsin treatment efficiently cleaves RBC membrane glycoproteins—notably glycophorin A, a sialoglycoprotein responsible for carrying ~75% of RBC membrane-associated sialic acid residues (*54*–*56*). Trypsin-treated RBCs, like neuraminidase-treated RBCs, exhibited a decreased zeta potential magnitude (i.e., less negative and more neutral) after cleavage of membrane glycoproteins carrying negatively charged residues (fig. S3C) (*12*, *57*). Trypsin treatment reduced PACE60 nanoparticle adsorption efficiency by an average of 66%, reduced the proportion of RBCs with associated nanoparticles by an average of 61%, and reduced the relative number of nanoparticles per RBC by an average of 37% (Fig. 4). These findings suggest that RBC membrane sialic acid quantity is positively correlated with PACE60 nanoparticle adsorption efficiency.

As sialic acid quantity is the primary determinant of RBC surface charge, we questioned whether electrostatic interactions between cationic PACE60 nanoparticles and the negatively charged RBC surface were driving these findings. Two additional experiments were carried out that indirectly assessed whether electrostatic potential modulation influenced PACE60 nanoparticle adsorption, providing potential mechanistic context to these findings. In both experiments, nanoparticle zeta potential (as measured in water) was reduced by either the introduction of murine plasma (presumably forming an anionic protein corona around the nanoparticles) or by altering the cargo encapsulated within the nanoparticles (incorporation of an increased molar quantity of double-stranded DNA, thereby increasing the negatively charged phosphate backbone content by 227% as compared to the ssDNA-encapsulated formulation). Both of these experiments demonstrated a reduction in nanoparticle adsorption efficiency (figs. S4 and S5, respectively).

However, it should be noted that the zeta potential of PACE60 nanoparticles in the adsorption suspension medium (DPBS) was near neutral even in the absence of these modulations (fig. S5, C and D)—presumably due to the high ionic strength of DPBS as compared to water, allowing more robust screening of charges at the nanoparticle’s surface by negative counterions. Furthermore, these modulations (introduction of murine plasma or encapsulation of more negative cargo) may have changed more than nanoparticle zeta potential. For instance, plasma exposure also produced an increase in nanoparticle size (fig. S4, E and F), and the resultant protein corona may exhibit an altered propensity to form nonelectrostatic bonds with the RBC membrane. In addition, trypsin treatment may cleave other surface proteins in addition to sialoglycoproteins. These findings demonstrate the correlative limitations of extending modulated variables to their proposed mechanism, although findings remain meaningful from mechanistic hypothesis generation and manufacturing perspectives.

### RBC hitchhiking increases PACE60 nanoparticle exposure to murine visceral organs, particularly the lung

Through the above experiments, we have confirmed that PACE60 nanoparticles can be adsorbed onto RBCs in vitro. Using PACE60 nanoparticles as a model system, we have also shown that cellular properties correlated with the storage lesion are determinants of nanoparticle-RBC adsorption efficiency. To demonstrate the utility of RBC hitchhiking as a PACE60 nanoparticle administration strategy, we conducted biodistribution assessments for two PACE60 nanoparticle formulations—one encapsulating a hydrophobic small molecule (DiD) and one encapsulating an oligonucleotide (Cy5-ssDNA)—using the information gathered regarding optimized adsorption parameters described above.

**Fig. 5.**
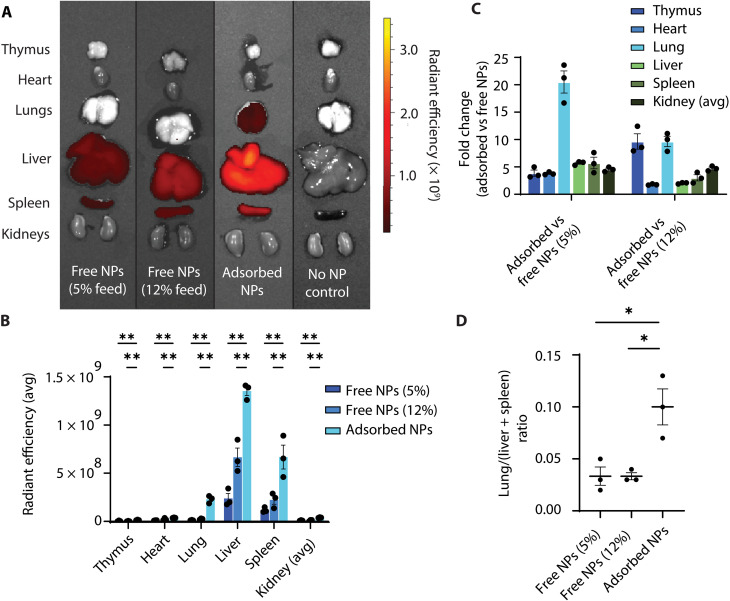
Biodistribution evaluation of RBC hitchhiking PACE60 nanoparticles encapsulating DiD. (**A**) Composite image of representative IVIS images displaying radiant efficiency [(photons/s/cm^2^/sr)/(μW/cm^2^)] following administration of hitchhiked versus free PACE60 nanoparticles encapsulating DiD. Feed dose is the total dose of nanoparticles added to RBCs prior to adsorption incubation and postadsorption washes. Color scale: minimum = 9.00 × 10^7^, maximum = 3.50 × 10^9^. (**B**) Quantification of organ average radiant efficiency following administration of hitchhiked versus free PACE60-DiD nanoparticles. Experimental *n* = 3, control *n* = 2. (**C**) Fold changes of organ radiant efficiencies of hitchhiked versus free PACE60-DiD nanoparticle group averages; *n* = 3. (**D**) Lung/(liver + spleen) ratio of average radiant efficiency of animals administered hitchhiked versus free PACE60-DiD nanoparticles. *P < 0.05; **P < 0.01. Lines and error bars in individual data point plots indicate means ± SEM. PACE60, poly(amine-*co*-ester) synthesized with 60 mol % PDL; IVIS, in vivo imaging system; NP, nanoparticle; RBC, red blood cell; avg, average.

Administration of RBC-adsorbed PACE60 nanoparticles encapsulating DiD enhanced cargo exposure to visceral organs several-fold as compared to free nanoparticles delivered at a dose of 3 mg/kg (≈5% of adsorption feed dose) and assessed 14 hours postadministration. The largest increase in delivery efficiency was observed in the lung (21-fold increase), consistent with previously reported trends with other polymeric nanoparticles exhibiting increased deposition in the first microcapillary bed encountered after intravascular administration. Thymus, heart, liver, spleen, and kidneys were also assessed. These organs exhibited a three- to sixfold increase in hydrophobic cargo deposition (Fig. 5, A to C, and fig. S6). Mice were perfused with 20 ml of heparin solution in DPBS (100 U/ml; >10x blood volume) via cardiac puncture immediately following euthanasia and exsanguination, decreasing the likelihood that residual RBCs with associated nanoparticles contributed meaningfully to measured fluorescence. As hepatosplenic deposition was the most likely outcome for free nanoparticles administered intravenously, lung-to-(liver + spleen) signal ratios were compared, suggesting significantly increased delivery efficiency to pulmonary tissues via RBC hitchhiking relative to hepatosplenic tissues (Fig. 5D).

Earlier, we confirmed the linearity of our in vitro adsorption efficiency assay and ensured correlation with an alternative measurement modality (fig. S7). Still, we performed an additional, more conservative comparison of the biodistribution of free versus RBC hitchhiking PACE nanoparticles to ensure enhancements in the biodistribution profile seen in the hitchhiking group were due to the adsorption offloading mechanism as opposed to an undetected dosing discrepancy due to inaccuracy of the adsorption efficiency assay (Fig. 1E). Increasing the dose of free DiD-containing nanoparticles delivered to 8 mg/kg (≈12% adsorption feed dose, the highest adsorption efficiency measure observed) still enabled superior detection of DiD cargo in visceral organs with disproportionately high pulmonary delivery efficiency, with average fold increases of 2 to 10 detected for imaged organs (Fig. 5, A to D, and fig. S6).

**Fig. 6.**
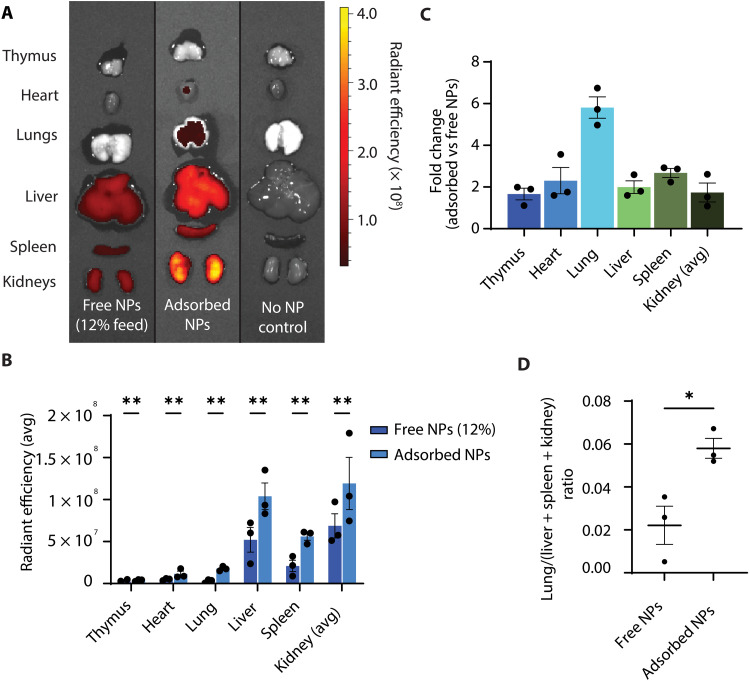
Biodistribution evaluation of RBC hitchhiking PACE60 nanoparticles encapsulating Cy5-ssDNA. (**A**) Composite image of representative IVIS images displaying radiant efficiency [(photons/s/cm^2^/sr)/(μW/cm^2^)] following administration of hitchhiked versus free PACE60 nanoparticles encapsulating Cy5-tagged ssDNA. Feed dose is the total dose of nanoparticles added to RBCs prior to adsorption incubation and postadsorption washes. Color scale: minimum = 3.18 × 10^7^, maximum = 4.10 × 10^8^. (**B**) Quantification of organ average radiant efficiency following administration of hitchhiked versus free PACE60-ssDNA nanoparticles. Experimental *n* = 3, control *n* = 1. (**C**) Fold changes of organ radiant efficiencies of hitchhiked versus free PACE60-ssDNA nanoparticle group average; *n* = 3. (**D**) Lung/(liver + spleen + kidney) ratio of average radiant efficiency of animals administered hitchhiked versus free PACE60-ssDNA nanoparticles. *P < 0.05; **P < 0.01. Lines and error bars in individual data point plots indicate means ± SEM. PACE60, poly(amine-*co*-ester) synthesized with 60 mol % PDL; IVIS, in vivo imaging system; ssDNA, single-stranded DNA; NP, nanoparticle; RBC, red blood cell; avg, average.

Comparing the biodistribution of adsorbed versus free nanoparticles encapsulating Cy5-ssDNA at this conservative dosage comparison (free nanoparticle dose = 11 mg/kg ≈ 12% adsorption feed dose) also demonstrated enhanced visceral organ exposure with a disproportionately high efficiency of pulmonary delivery (sixfold versus two- to threefold; Fig. 6, A to D, and fig. S8). Again, murine vasculature was perfused prior to organ procurement, and Cy5 transfer to RBCs during the adsorption process was confirmed to be minimal (fig. S1C). Substantial kidney cargo deposition was observed in both groups, potentially due to the glomerular filtration of free Cy5-ssDNA or free Cy5 following release from nanoparticles.

Of note, the half-life of free PACE60 nanoparticles delivered at a similar dose was previously reported to be on the order of minutes (<15 min) (*1*); thus, 14 to 16 hours was selected for these experiments to enable near-complete distribution of circulating nanoparticles while still enabling sufficient sensitivity of signal detection prior to cargo metabolism. Pharmacokinetic analysis of blood specimens collected 30 s after nanoparticle administration showed no significant difference in the particle dose administered between experimental groups. Furthermore, analysis of blood specimens collected 16 hours postadministration showed no significant difference in the particle dose remaining in circulation at time of organ procurement between experimental groups. The mean percentage of particles remaining in circulation at time of organ procurement was slightly higher in the adsorbed nanoparticle group (4% versus 1%); however, this finding was not statistically significant (fig. S9). These findings support the interpretation that doses delivered between these two groups were equivalent, and adequate time was provided for near-complete distribution of doses in both experimental groups.

In summary, intravenous administration of RBC-adsorbed PACE60 nanoparticles increased biodistribution to visceral murine organs, particularly the lung, as measured 14 to 16 hours postinjection, as compared to free nanoparticles at equivalent doses.

## DISCUSSION

PACE nanoparticles exhibit a reduced toxicity profile relative to other more heavily cationic polymeric nanoparticles (*34*), a well-characterized murine PK/BD profile (*1*), and a demonstrated ability to encapsulate both hydrophobic small molecules (*1*) as well as oligonucleotides (*1*, *39*) and mRNA (*37*, *44*). Intravenously administered free PACE nanoparticles with 60% PDL composition predominately accumulate in the liver and spleen (*1*). RBC hitchhiking offers a strategy to modulate this biodistribution profile, enabling enrichment in pulmonary tissue and improved delivery to other visceral organs.

Although doses are limited by safe transfusion and relative red cell volumes, nanoparticle doses can be tuned by increasing the NP:RBC ratio of the adsorption feed dose to the point of adsorption saturation or deleterious RBC health (e.g., aggregation or hemolysis). These limits were not reached in this study using PACE60 nanoparticles at doses on par with those previously reported for oligonucleotide delivery in preclinical animal disease models (*39*). Adsorption at a 100:1 NP:RBC feed ratio did not substantially affect aggregation or hemolysis. PACE60 nanoparticle-RBC complexes appear stable during 4°C storage over at least 30 hours, enabling manufacturing flexibility. However, a fair degree of variability was observed in adsorption efficiency over the entire study period, highlighting the need for precision during future protocol development.

Despite the interest in using cells as carriers for nanoparticle drug delivery systems (*4*), cellular determinants of polymeric nanoparticle adsorption efficiency have rarely been reported in the literature. The adsorption efficiency of PLGA and PLGA-PEI nanoparticles to RBCs of multiple species has been studied, enabling a direct comparison of how cell source can influence the adsorption of the same nanoparticle formulation. These findings demonstrated significant cross-species differences in adsorption efficiency (*25*). Biomaterial-glycocalyx interactions have been identified as potential determinants of drug delivery vehicle uptake in general; their impact on RBC hitchhiking has been reported (*58*–*60*) but for gold nanoparticles with an organic ligand shell, a system quite different in size, composition, density, and other properties shared by most polymeric nanoparticle systems (*12*). Additional investigations into cellular determinants of the adsorption efficiency of polymeric nanoparticles are lacking.

This study demonstrated the deleterious effect the storage lesion can have upon PACE60 nanoparticle adsorption to murine RBCs. This finding is important to consider in the design of future RBC hitchhiking studies in animal models and highlights the need for future studies using human RBCs stored in various Food and Drug Administration–approved anticoagulant/preservative and additive solutions used in the blood component manufacturing industry (*25*, *61*). Decisions regarding the use of allogeneic, off-the-shelf, donor RBCs versus autologous RBCs and the consequent logistical implications will likely influence the specifics of such studies.

The storage lesion is a composite phenomenon composed of many variables including membrane stiffness, glycocalyx composition, zeta potential, RBC morphology, and metabolic by-products found in stored RBCs and their suspension mediums (*28*). RBC stiffness, as modulated by adsorption temperature or RBC fixation, variably affected adsorption efficiency. Glutaraldehyde fixation has been shown to decrease RBC deformability and increase the RBC membrane Young’s modulus (*50*). A previous study has reported that RBC membranes envelope, or “cup,” the contacting aspect of adsorbed polystyrene nanoparticles, suggesting that RBC membrane deformability is a potential determinant of adsorption efficiency (*9*). Furthermore, fixation has been shown to inhibit polystyrene nanoparticle adsorption and induce the detachment of polystyrene nanoparticles from RBCs postadsorption (*9*, *20*). Fixation was also shown to inhibit PACE60 nanoparticle adsorption in this present study, suggesting that RBC membrane stiffness is an important cellular determinant of adsorption efficiency.

However, this relationship is more complex than we initially anticipated. RBCs exhibit increased measures of, and correlates to, stiffness (e.g., elastic modulus, shear modulus, deformability, etc.) at lower temperatures, although not to the same magnitude as is induced by fixation (*47*–*50*). Adsorption at colder temperatures was associated with counterintuitively higher nanoparticle retention. Although the effect of temperature on the RBC membrane was likely not an isolated change—nanoparticle biomechanical properties and the adsorption system’s entropy, governing nanoparticle-RBC interaction and offloading probabilities, are also likely influenced by reduced temperatures—this finding does suggest that the magnitude of the effect that membrane stiffness has upon adsorption efficiency may be modulated through interaction with other variables in the system. As this relationship is not completely linear, additional studies with human RBCs will be needed prior to clinical translation. Several donor phenotypes have previously been associated with decreased RBC stiffness (*31*, *62*–*65*). Regardless of whether off-the-shelf donor RBCs or autologously collected RBCs are used for clinical translation, interactions between nanoparticle materials, adsorption temperature, and prevalent stiffness-associated donor/patient phenotypes should be evaluated. These findings are also important to consider when developing RBC adsorption protocols to ensure efficient parameters are chosen, particularly in light of the variable adsorption temperatures used in prior RBC hitchhiking studies.

Our findings also demonstrate the importance of RBC membrane-bound sialoglycoprotein quantity as a determinant of nanoparticle-RBC adsorption efficiency. Trypsin-treated RBCs having drastically fewer membrane-associated sialic acid residues were less efficient substrates for PACE60 nanoparticle adsorption. RBC sialic acid content decreases as a function of storage duration (*30*, *66*). In addition, donor phenotype has been shown to influence sialic acid quantity. For instance, RBCs from donors with type 2 diabetes exhibit an altered sialidase activity profile and consequent modulation in membrane sialic acid content (*57*, *65*, *67*). Elucidating the effects of storage and donor phenotype variables upon sialic acid content and, consequently, nanoparticle adsorption will be essential prior to clinical translation of the RBC hitchhiking strategy.

The mechanism by which sialic acid residues facilitate PACE60 nanoparticle adsorption remains unclear. Additional experiments reducing PACE60 nanoparticle surface charge by the incorporation of a negatively charged nanoparticle protein corona or the encapsulation of an increased quantity of negatively charged cargo provided correlative evidence that electrostatic interactions may be mechanistically at play. However, as the zeta potential of PACE60 nanoparticles is near neutral when measured in DPBS (the adsorption suspension medium), such may not be the driving force that initiates nanoparticle-RBC binding. Electrostatic interactions may merely stabilize adsorption once the proximity between both surfaces has been reduced and negatively charged counterions in solution have been excluded. Many other nanoparticle materials have been adsorbed to the surface of RBCs, several of which are anionic. As such, other noncovalent forces such as hydrophobic interactions, van der Waals forces, or hydrogen bonding may drive these interactions. However, prior studies have demonstrated that the physiochemical properties governing surface charge may be more important than zeta potential (i.e., electrical potential at the slipping plane) with regard to nanoparticle-cell interactions (*68*, *69*). Additional investigations regarding these interactions are warranted.

Previous studies have demonstrated that intravascular delivery of RBC hitchhiking nanoparticles generally results in deposition enrichment in the next-in-circuit microcapillary bed via a “first-pass” effect [e.g., the lungs following intravenous delivery; (*9*, *11*, *16*–*20*, *23*, *24*, *26*)]. The biodistribution of intravenously administered RBC hitchhiking PACE60 nanoparticles also followed this principle, with a 5- to 19-fold increase lung delivery observed. All nonpulmonary organs assayed also exhibited an increase in cargo detection following administration of RBC-hitchhiked PACE60 nanoparticles compared to free nanoparticles. This includes delivery to the liver and spleen, the primary organs responsible for free PACE60 nanoparticle uptake. Two doses of free nanoparticles, one reflective of the likely dose delivered via hitchhiking and one at the conservative upper limit of that estimation, were used in comparator groups across two nanoparticle formulations to ensure that dosing measurement inaccuracies were not the cause of these increased delivery measurements. At the time of organ procurement, the percentage of nanoparticle cargo remaining in circulation was slightly higher in the hitchhiked group, although this difference was small and not statistically significant. Both groups demonstrated near-complete (>95%) nanoparticle deposition at this time. It is possible that RBC hitchhiking shifted cargo delivery from organs that were not assayed (e.g., gastrointestinal tract, musculature, skin, etc.) to organs that were. However, it is also possible that the kinetics of nanoparticle retention, cargo metabolism, and/or cargo fate are also altered via hitchhiking, as has been suggested in previous reports (*9*, *22*, *23*). Nonetheless, it is clear that RBC hitchhiking increases delivery of PACE60 nanoparticle cargo to various organs, particularly the next-in-circuit organ, as examined at time points that are numerous free-nanoparticle half-lives after administration (*1*).

Limitations of this study include the use of fluorescent modalities for nanoparticle tracking, limiting the sensitivity of detection relative to other modalities such as radiolabeled cargo. Cargo dissociation from nanoparticle encapsulation, particularly as may have been observed in the biodistribution evaluation of Cy5-ssDNA–encapsulating nanoparticles, also makes it challenging to deconvolute nanoparticle-plus-cargo from free cargo delivery, although such would reflect cargo delivery regardless. In addition, many cell treatments used in the reported experiments provided indirect modulation of cell characteristics and thus correlative evidence of adsorption efficiency modulation when extended to proposed mechanisms. Last, as there are strain-specific differences in murine RBC storage kinetics, with these kinetics differing from those observed in human RBCs, experimentation using human RBCs will ultimately be necessary.

This study has demonstrated that PACE60 nanoparticles can be adsorbed to RBCs in a robust and reproducible manner without causing substantial deleterious effects to RBC health. Cellular determinants, namely, the storage lesion and correlated variables of stiffness and cell surface sialic acid quantity, also dictate the adsorption efficiency of PACE60 nanoparticles and will be important variables to consider when studying RBC hitchhiking using non-PACE nanoparticle systems as well as during clinical translation of the RBC hitchhiking strategy. Last, RBC hitchhiking can be used to increase PACE60-mediated delivery of hydrophobic small molecules or nucleic acid cargoes to target organs.

## MATERIALS AND METHODS

This section describes the materials and methods used to evaluate whether PACE60 nanoparticles can be nonspecifically adsorbed to RBCs, assess whether cellular variables associated with the RBC storage lesion influence the efficiency of this interaction, and determine whether and how PACE60 nanoparticle biodistribution can be modulated by using an RBC hitchhiking strategy.

### Materials

Materials used can be found tabulated in table S2.

### PACE synthesis and purification

PACE polymers were synthesized and characterized as previously described (*38*). Briefly, PDL, *N*-methyldiethanolamine, and diethyl sebacate were copolymerized via a Novozym 435 enzyme catalyst in diphenyl ether. Sixty percent PDL (mol %) was used for all PACE polymers (PACE60). Two reaction phases were carried out: oligomerization at 90°C under 1 atm (101.325 kPa) of argon gas for 20 hours followed by polymerization at 90°C under vacuum for 48 hours. After synthesis, the diphenyl ether solvent was removed via hexane washes, and the PACE polymer was dissolved in dichloromethane (DCM) or chloroform and filtered. Solvents were removed using a rotary evaporator, and polymers were stored at −20°C.

### Nanoparticle formulation and characterization

PACE60 nanoparticles were formulated using a double (water-in-oil)-in-water emulsion solvent evaporation technique as previously described (*1*). Briefly, 50 mg of polymer was dissolved in DCM to a volume of 1 ml overnight. If encapsulated, DiD in dimethyl sulfoxide was added to the dissolved polymer immediately prior to formulation [0.5% (w/w)]. Oligonucleotide cargo (44 to 50 nmol) in 100 to 150 μl of Tris-EDTA buffer (or H_2_O for DiD or unloaded particles) was added dropwise under vortex to the polymer (± dye) solution and sonicated with a probe tip sonicator to form a water-in-oil emulsion. The water-in-oil emulsion was added dropwise under vortex into 2 ml of 5% (w/v) low–molecular weight poly(vinyl alcohol) (PVA) solution in H_2_O and sonicated with a probe tip sonicator to form a (water-in-oil)-in-water emulsion and then diluted into 10 ml of 0.3% (w/v) PVA in H_2_O while mixing. The remaining organic solvent was evaporated using a rotary evaporator. Nanoparticles were then washed twice in H_2_O by centrifugation to remove excess PVA. PACE60 nanoparticles were characterized as below and flash frozen in liquid nitrogen for storage at −80°C. One aliquot was lyophilized to obtain mass/concentration.

PLGA nanoparticles were formulated using a single emulsion-solvent evaporation technique. Ester-terminated PLGA polymer with a 50:50 lactide:glycolide ratio and molecular weight 54,000 to 69,000 g/mol was commercially acquired. The polymer was dissolved at 50 mg/ml in DCM overnight at room temperature. The polymer solution was sonicated for 30 s in an ultrasound bath and vortexed. This polymer solution was added dropwise to 2 ml of 5% (w/v) PVA (molecular weight 13,000 g/mol) solution while vortexing. This mixture was ultrasonicated three times for 10 s each using a probe tip sonicator to form an oil-in-water single emulsion. This resulting single emulsion was diluted into 10 ml of PVA solution at 0.3% (w/v) in a beaker stirred at 400 rpm. Then, a flask with this mixture was placed on a rotary evaporator for 15 min at 80 mbar at room temperature to allow solvent evaporation. The nanoparticles were centrifuged at 18,000 RCF and 4°C for 45 min and then washed twice in ultrapure water by centrifugation to remove excess PVA. After the final centrifugation, the supernatant was removed, and the nanoparticles were resuspended at a concentration of 5 mg/ml in water. The nanoparticles were aliquoted, flash frozen in liquid N_2_, and stored at −80°C.

Polystyrene nanoparticles were acquired from a commercial source and stored at 4°C.

Nanoparticle size and zeta potential were measured in triplicate via DLS using a Malvern Instruments Zetasizer Nano-ZS. Nanoparticles were sonicated prior to use to minimize aggregation and were never refrozen for later use. A summary of nanoparticle formulation specifications and resulting characteristics can be found in table S1. DLS data demonstrate measurement replicates unless batch replicates are noted.

Data demonstrating nanoparticle storage stability are presented in fig. S10. Nanoparticles demonstrated a slight increase in diameter and polydispersity index following freeze/thaw that was largely stable over prolonged storage. Data demonstrating serum stability are presented in fig. S11. For the latter experiments, PACE60 nanoparticles were exposed to 95% mouse serum for various time intervals and subsequently diluted into 10% mouse serum in water for DLS measurements. As measurements were confounded by the presence of plasma proteins/aggregates, the diameter (intensity derived) of the dominant peak on the size distribution is reported, along with the size of the peak itself on the distribution.

### RBC treatments and characterization

All animal procedures were performed in accordance with the policies of the Yale Animal Resources Center and approved by Yale University’s Institutional Animal Care and Use Committee (ID 11228). RBCs were procured via retro-orbital exsanguination of isoflurane-euthanized wild-type male and female C57BL/6 mice from Charles River. RBCs were collected in 15% CPDA-1, leukoreduced via filtration, and volume reduced to 70% hematocrit for storage at 4°C in 1.5-ml microcentrifuge tubes (*70*). RBCs were used within 24 hours unless otherwise noted.

For confocal imaging, RBCs were labeled with Vybrant DiO prior to adsorption (5 μM, 1 hour at 37°C, 16 hours at 4°C). For experiments requiring flow cytometric endpoints, RBCs were labeled with anti-(murine RBC)–fluorescein isothiocyanate (FITC) conjugates (clone TER-119; 7.1 μg/ml, 30 min at ambient temperature, 16 hours at 4°C) prior to absorption. Isotype-FITC controls confirmed minimal degree of nonspecific staining. For experiments requiring neuraminidase treatment, neuraminidase in DPBS (-Ca, -Mg for all instances of use here) was incubated with RBCs (5% hematocrit) at 0.2 U/ml at ambient temperature for 30 min, 37°C for 3.5 hours, and 0.8 U/ml at 37°C for 1 hour. For experiments requiring trypsin treatment, RBCs were incubated with trypsin-EDTA (0.25%) at 37°C for 16 hours followed by three DPBS washes. Untreated RBCs were exposed to the same conditions without trypsin. For experiments requiring glutaraldehyde fixation, RBCs (10% hematocrit) were fixed in 0.25% glutaraldehyde in DPBS on ice for 30 min, washed 3x in DPBS, and then stored at 4°C for 16 hours.

RBC zeta potential measurements were carried out via DLS using a Malvern Zetasizer Nano-ZS. Specimens were measured at a hematocrit of 0.007 to 0.07% in DPBS.

### Nanoparticle-RBC adsorption

RBCs were washed three times in DPBS prior to adsorption. Nanoparticles (stock concentrated in water) were added to RBCs in DPBS on ice at a hematocrit of 5% and an NP:RBC ratio of 100:1 unless otherwise noted. Water introduction to the adsorption suspension was minimized such that tonicity of the final adsorption environment was not altered by >5%. Nanoparticles and RBCs were incubated together for 1 hour, mixing every 15 min. Specimens were centrifuged at 100 RCF (low volume, microcentrifuge tubes) or 500 RCF (high volume, 15-ml conical vials) for 5 min, and the supernatant was removed. Three additional high-volume DPBS washes were carried out to ensure removal of nonadsorbed nanoparticles unless otherwise noted. Adsorbed nanoparticle-RBC complexes were used within 12 hours unless otherwise noted.

### Nanoparticle-RBC adsorption characterization

Confocal imaging was performed using a Zeiss LSM 880 with Airyscan. Following adsorption, 5 μl of specimen was deposited onto a 14-mm-window, number 1.5 glass-bottom 35-mm dish and compressed with gravity using a 10-mm, number 1.5 glass coverslip. Nanoparticle-RBC complexes were imaged with an axial stage movement of 128 nm. Images were processed using the Fiji distribution of ImageJ (*71*). Representative image processing consisted of global, channel-specific background subtraction as measured by average pixel intensity of background regions of interest, noise reduction using a 3 x 3 median filter, and channel-specific brightness/contrast adjustments to optimally display the spatial relationship between nanoparticle and RBC channels. FOVs displayed in movie S1 were processed via channel-specific brightness/contrast adjustments only. Manders colocalization coefficients quantifying the proportion of nanoparticle signal exhibiting overlap with RBC signal were derived using the JACoP plugin on images that were processed via background subtraction as above, Li-algorithm thresholding, and noise reduction as above (*72*).

Adsorption efficiency was defined as the percentage of fluorescence signal remaining following postadsorption washes over signal present prior to supernatant removal and washes. Specimens were frozen and thawed to lyse RBCs prior to measurement. Fluorescence was measured via fluorimetry of specimens deposited into 384-well clear, flat-bottom plates and carried out on an EVOS FL Auto 2 Cell Imaging System with a Cy5 filter and an Olympus super-apochromat 20x/0.75–numerical aperture objective using a technique adapted from a previous report (*73*). The most inferior plane in which settled nanoparticles could be identified was chosen as the focal plane. Nine FOVs were imaged per well/specimen. Background was derived from averages of FOV pixel sums corresponding to no-nanoparticle controls and subtracted from experimental group FOV pixel sums. Each set of nine background-corrected FOV pixel sums were averaged, and percentages remaining for each specimen were determined. Linearity was confirmed within the range of possible nanoparticle concentrations used. Results obtained via microscope fluorimetry measures were compared with those obtained from a Molecular Devices SpectraMax M5 plate reader fluorimeter (fig. S7).

Flow cytometry was carried out on a BD LSR II or a Cytek Aurora. Gating strategies are described in the Supplementary Materials (figs. S12 and S13).

Hemolysis was quantified via measuring the 550-nm absorbance of the nanoparticle-RBC adsorption supernatant prior to postadsorption washes using a Molecular Devices SpectraMax M5 multimodal plate reader similar to previously reported assays (*8*, *13*, *14*, *17*, *24*, *25*). Linearity was confirmed via linear regression of a standard curve derived from osmotically lysed RBC solutions (fig. S14).

### In vivo nanoparticle administration and biodistribution/pharmacokinetic analyses

Nanoparticle-RBC complexes were administered to wild-type, 8- to 12-week-old female C57BL/6 mice from Charles River via retro-orbital injection of 150 μl at 50% hematocrit in DPBS through a 1-ml syringe and 27-gauge needle while under isoflurane anesthesia. Free nanoparticles were also administered diluted in DPBS. Approximately 30 s following administration of PACE60 nanoparticles encapsulating Cy5-ssDNA, 2 μl of blood was collected from a tail nick made with a sterile blade and deposited into 9 μl of heparin in DPBS (100 U/ml), adapting a procedure from previously published protocols (*73*). Nanoparticles were allowed to circulate for 14 to 16 hours prior to mouse euthanasia and organ procurement. At time of procurement, an additional 2 μl of blood was collected from mice administered PACE60 + Cy5-ssDNA nanoparticles. For all mice, maximal blood was collected via cardiac puncture, and 20 ml of heparin in DPBS solution (100 U/ml) was perfused through the vasculature from the cardiac puncture site. After 2 ml of perfusion, the inferior vena cava was cut to allow flushing of the remaining 18 ml of heparin perfusate. Thymus, heart, lung, liver, spleen, and kidneys were procured and stored on ice in 1 ml of DPBS. Organs were imaged via a PerkinElmer IVIS Spectrum optical imaging system. Average radiant efficiency was quantified for each organ for comparison. Signal fold changes of adsorbed versus free nanoparticle groups were calculated by dividing individual adsorbed organ radiant efficiencies by the average of those of the relevant free nanoparticle group.

### Statistical analyses

Statistical analyses were performed in GraphPad Prism 10. *t* tests were used to compare two groups of independent data. Paired *t* tests were used to compare two-groups of paired data. One-way or two-way analysis of variance (ANOVA) was used to compare more than two groups of data across one or two variables, respectively. Nonparametric test variants (e.g., Mann-Whitney test and Kruskal-Wallis test) were used in the setting of significant deviation from normality as detected by Prism’s suite of statistical normality tests (e.g., Shapiro-Wilk test). Welch’s correction was applied in the setting of heteroscedasticity. A significance level of 0.05 was used for all statistical tests throughout the study.
