# Supplementary material for: Cellular determinants influence the red blood cell adsorption efficiency of poly(amine-co-ester) nanoparticles
Source: Sci Adv. 2025 May 2;11(18):eadt8637. doi: 10.1126/sciadv.adt8637 (PMC12047439; doi:10.1126/sciadv.adt8637)
Supplement: Supplementary file 1 — Figs. S1 to S14 Tables S1 and S2 Legend for movie S1 Legend for data S1 [file sciadv.adt8637_sm.pdf]

Science Advances
AAAS

Supplementary Materials for

Cellular determinants influence the red blood cell adsorption efficiency of poly(amine-*co*-ester) nanoparticles

Thomas C. Binns *et al.*

Corresponding author: Thomas C. Binns, thomas.binns@yale.edu; W. Mark Saltzman, mark.saltzman@yale.edu

Sci. Adv. **11**, eadt8637 (2025)
DOI: 10.1126/sciadv.adt8637

The PDF file includes:

Figs. S1 to S14
Tables S1 and S2
Legend for movie S1
Legend for data S1

Other Supplementary Material for this manuscript includes the following:

Movie S1
Data S1

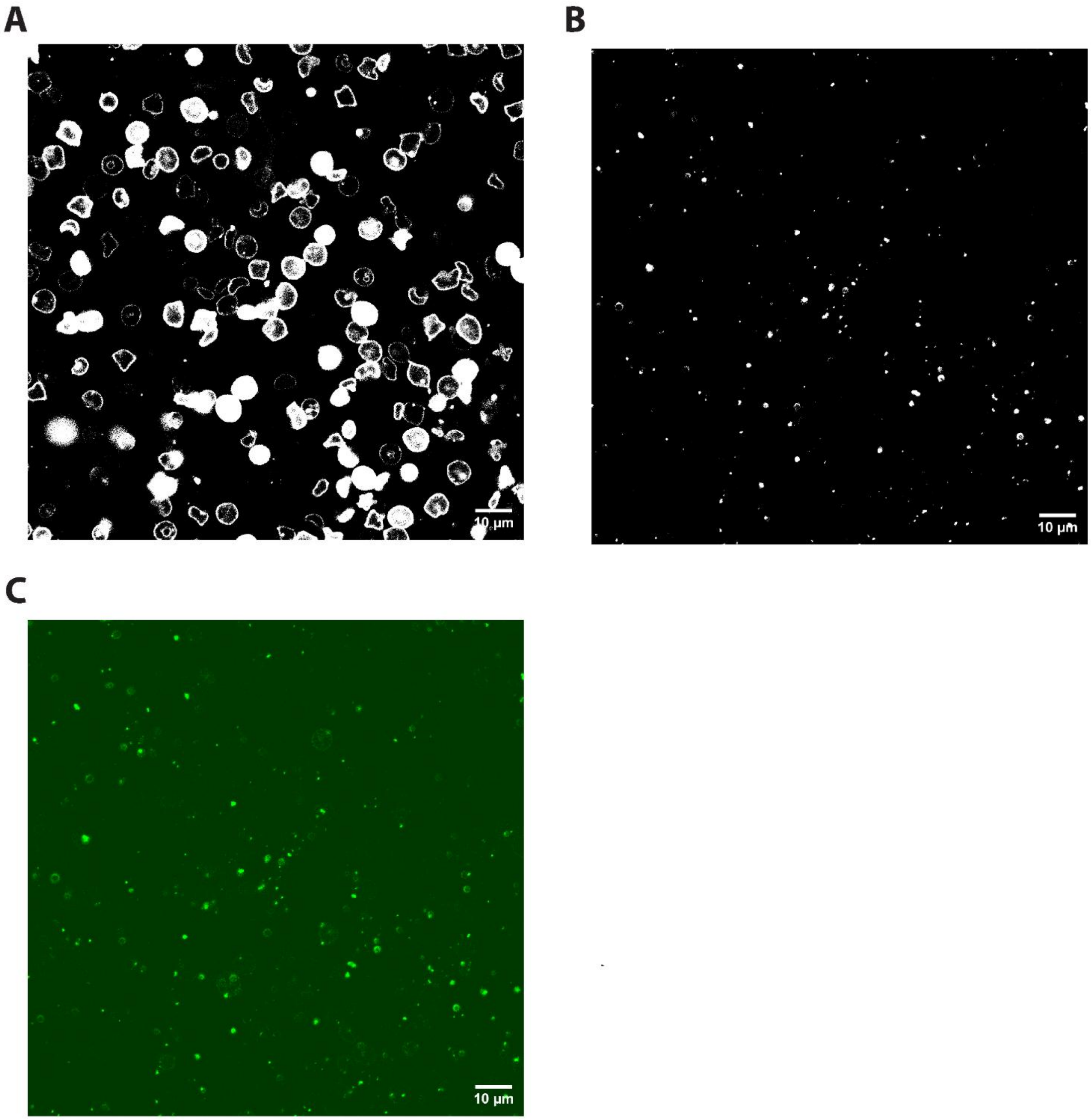

Fig. S1.

Confocal micrographs with various pre-processing applied. (A) Pre-processed DiO-labeled RBC channel utilized in Manders colocalization coefficient analysis along with (B) Pre-processed Cy5 nanoparticle channel utilized in Manders colocalization coefficient analysis. Pre-processing steps can be found in the *Materials and Methods* section. (C) Global contrast/brightness-pushed Cy5 nanoparticle channel utilized to demonstrate limited RBC morphology detection that could signify dye transfer from nanoparticles to RBCs. Scale bars = 10 µm. RBC = red blood cell.

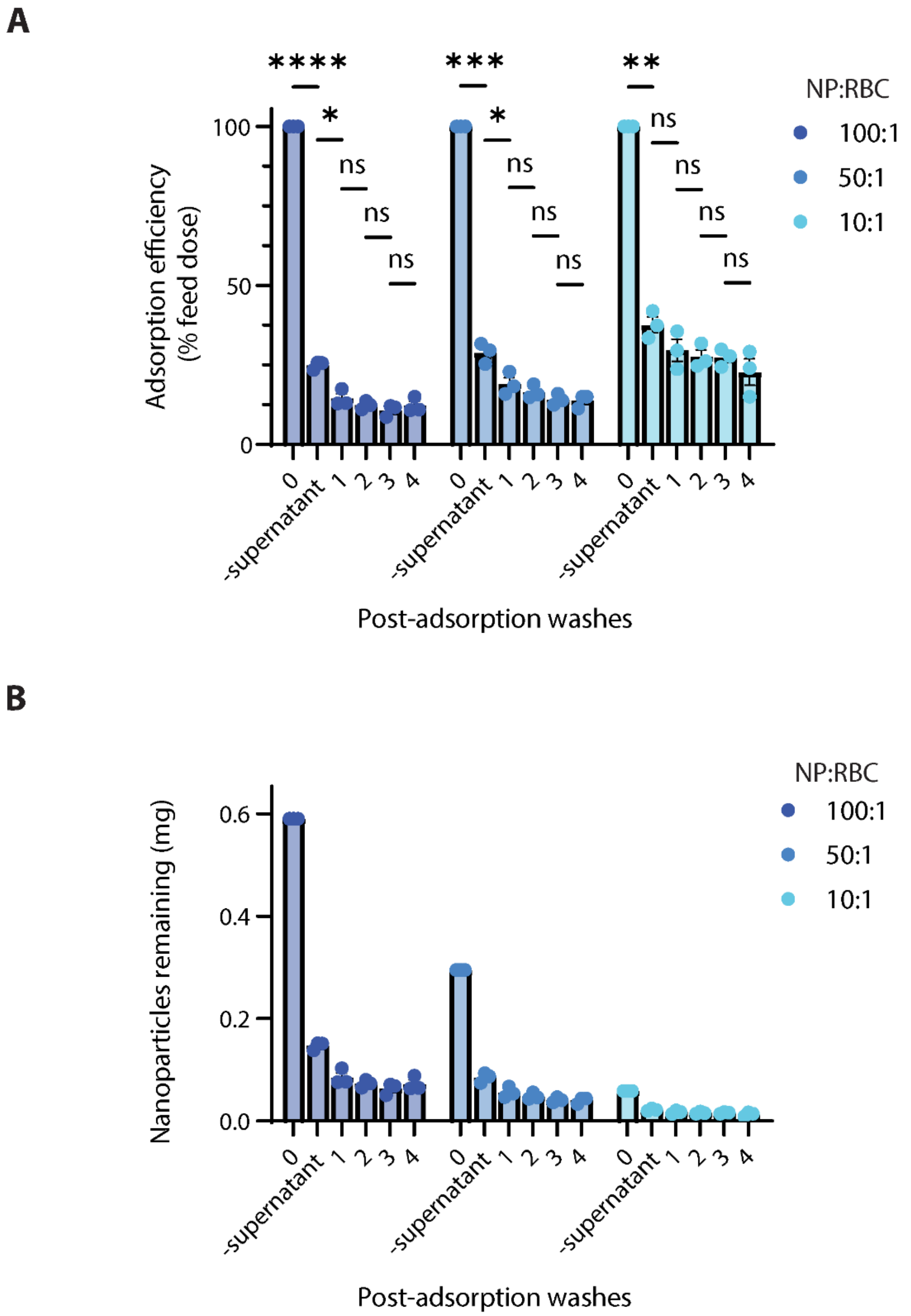

Fig. S2.

Investigation into the influence of nanoparticle:RBC ratio and post-adsorption washes upon (A) adsorption efficiency and (B) nanoparticle mass remaining. Data presented following 3 post-adsorption washes is representative of the standard adsorption protocol used throughout this study and is the same data presented in Fig. 1C, D. n = 3; * = $p < 0.05$, ** = $p < 0.01$, *** = $p < 0.001$, **** = $p < 0.0001$, ns = not significant. NP = nanoparticle, RBC = red blood cell.

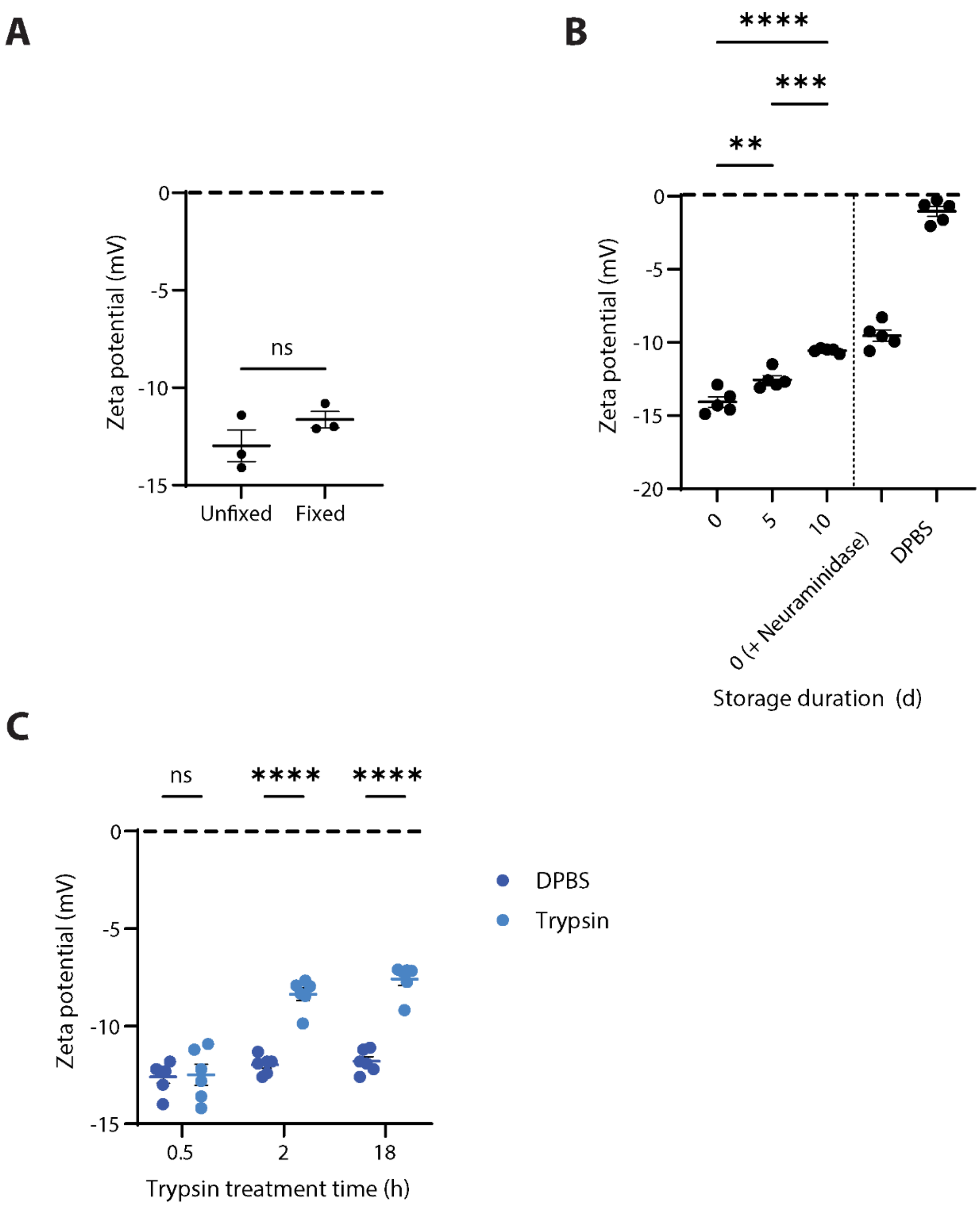

Fig. S3.

RBC zeta potential as a function of various treatments. (A) RBC zeta potential as a function of glutaraldehyde fixation. (B) RBC zeta potential as a function of storage duration. Neuraminidase treated RBCs are presented as a positive control. A DPBS control is presented as a negative control. (C) RBC zeta potential as a function of trypsin + EDTA treatment versus DPBS exposure time at 37 °C. n = 3-6 (measurement replicates); ** = $p < 0.01$, *** = $p < 0.001$, **** = $p < 0.0001$, ns = not significant. Line = mean ; error bars = standard error of the mean. RBC = red blood cell, DPBS = Dulbecco's Phosphate Buffered Saline (-Ca, -Mg), EDTA = ethylenediaminetetraacetic acid, d = days, h = hours.

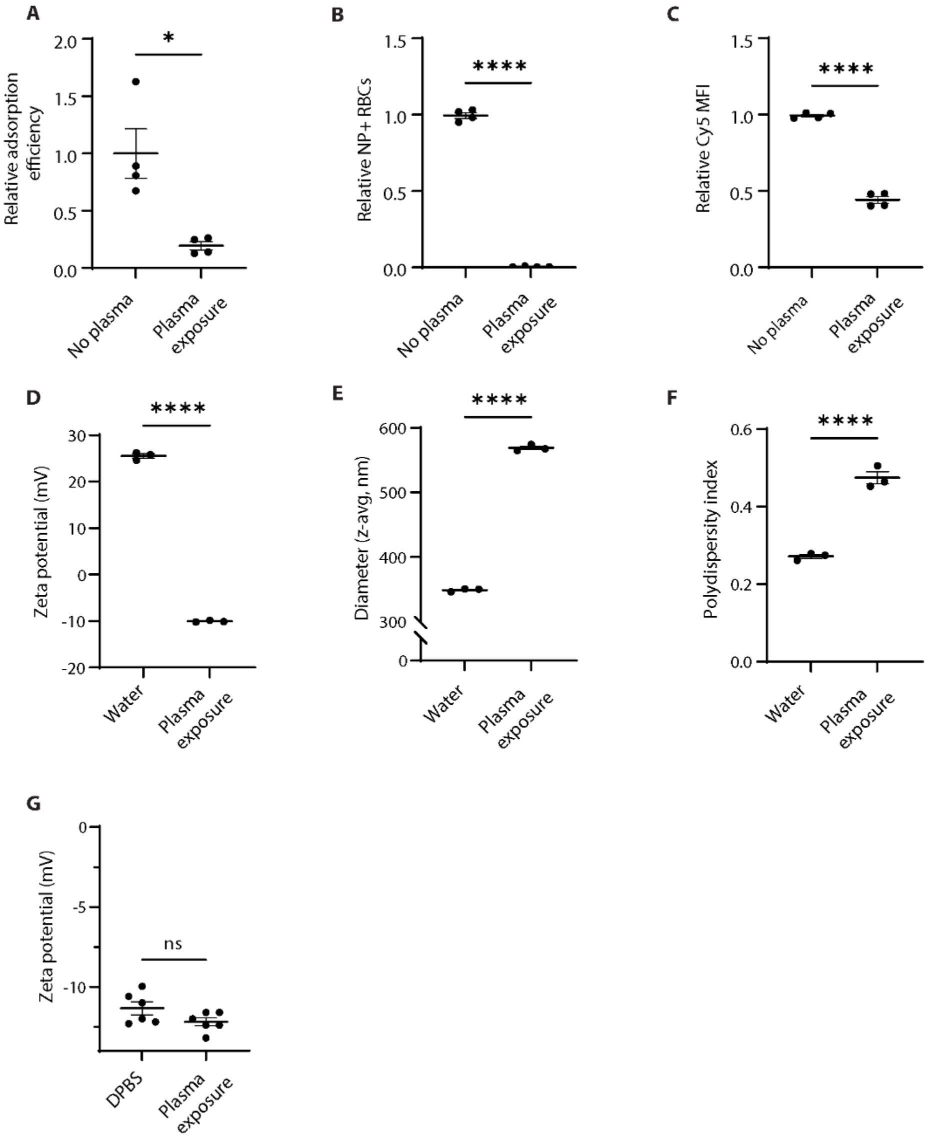

Fig. S4.

Adsorption efficiency, nanoparticle properties, and RBC zeta potential as a function of exposure to murine plasma (+ minority fraction of CPDA-1). (A) Relative adsorption efficiency of PACE60 nanoparticles, (B) relative percentage of RBCs with associated nanoparticle signal, and (C) relative median nanoparticle fluorescence intensity of nanoparticle positive cells as a function of plasma addition to the adsorption environment. n = 4. (D) Zeta potential, (E) diameter, and (F) polydispersity index measurements of nanoparticles in water as a function of plasma exposure. n = 3 (measurement replicates). (G) RBC zeta potential in DPBS as a function of plasma exposure. n = 6 (measurement replicates); * = $p < 0.05$, **** = $p < 0.0001$, ns = not significant. Lines and error bars indicate mean and standard error of the mean, respectively. RBC = red blood cell, CPDA-1 = citrate phosphate dextrose adenine anticoagulant solution, PACE60 = poly(amine-co-ester) synthesized with 60 mol% 15-pentadecanolide, DPBS = Dulbecco’s phosphate buffered saline (-Ca, -Mg), NP = nanoparticle, MFI = median fluorescence intensity.

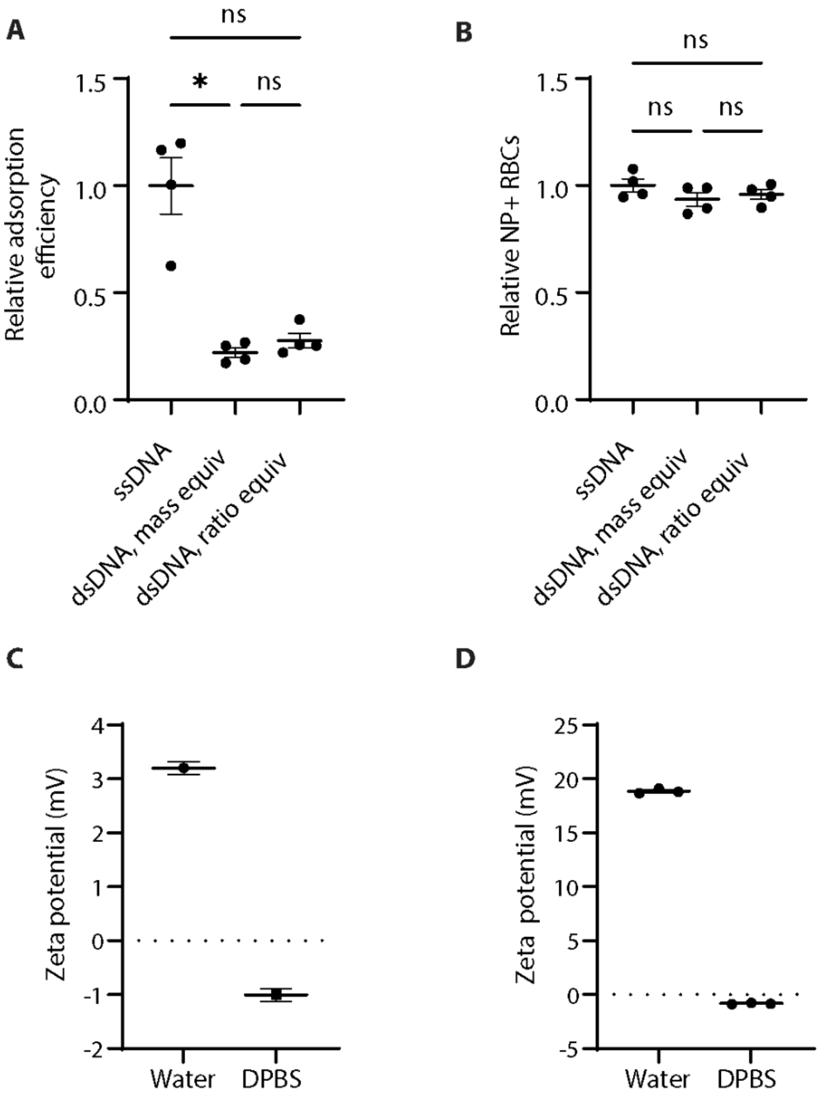

Fig. S5.

Adsorption efficiency and nanoparticle zeta potential as functions of differentially-charged cargo and measurement suspension medium, respectively. (A) Relative adsorption efficiency of PACE60 nanoparticles and (B) relative percentage of RBCs with associated nanoparticle signal as functions of differentially-charged nanoparticle cargos. Adsorptions of nanoparticles formulated with dsDNA evaluated via two groups equivalent in either number of nanoparticles or mass of nanoparticles. n = 4. Lines and error bars indicate mean and standard error of the mean, respectively. (C) Zeta potential of dsDNA-encapsulating PACE60 nanoparticles in water and DPBS. Only summary data is available; symbol/line = mean, error bars = standard deviation. (D) Zeta potential of ssDNA-encapsulating PACE60 nanoparticles in water and DPBS. n = 3 (measurement replicates); line = mean, error bars (visible but compressed) = standard deviation. * = $p < 0.05$, ns = not significant. RBC = red blood cell, PACE60 = poly(amine-co-ester) synthesized with 60 mol% 15-pentadecanolide, ssDNA = single-stranded DNA, dsDNA = double-stranded DNA, equiv = equivalent, NP = nanoparticle, DPBS = Dulbecco's phosphate buffered saline (-Ca, -Mg).

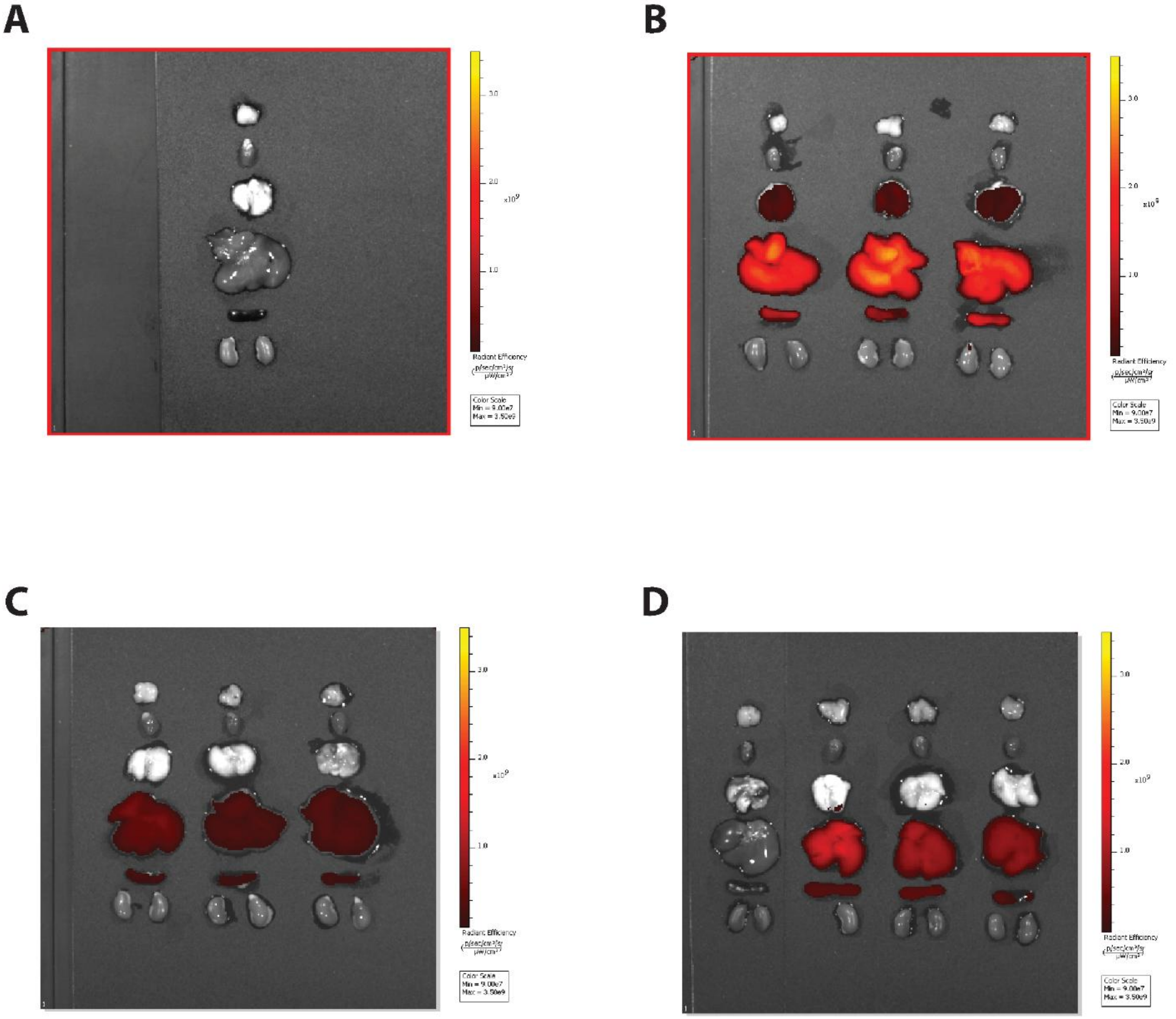

Fig. S6.

Full IVIS images displaying radiant efficiency [(photons/s/cm^2/sr)/(μW/cm^2)] from *in vivo* biodistribution experiments of PACE60 nanoparticles encapsulating DiD. Organs of mice from the following groups: (A) no-nanoparticle control #1, (B) hitchhiking group, (C) free nanoparticles at 5% adsorption feed dose group, (D) no-nanoparticle control #2 on left followed by free nanoparticles at 12% adsorption feed dose group. Experimental n = 3, control n = 2. Color scale: minimum = 9.00 x 10^7, maximum = 3.50 x 10^9. Organs shown top to bottom are: thymus, heart, lungs, liver, spleen, kidneys. Representative images of each group are duplicated in main text Figure 5A. IVIS = in vivo imaging system, PACE60 = poly(amine-co-ester) synthesized with 60 mol% 15-pentadecanolide.

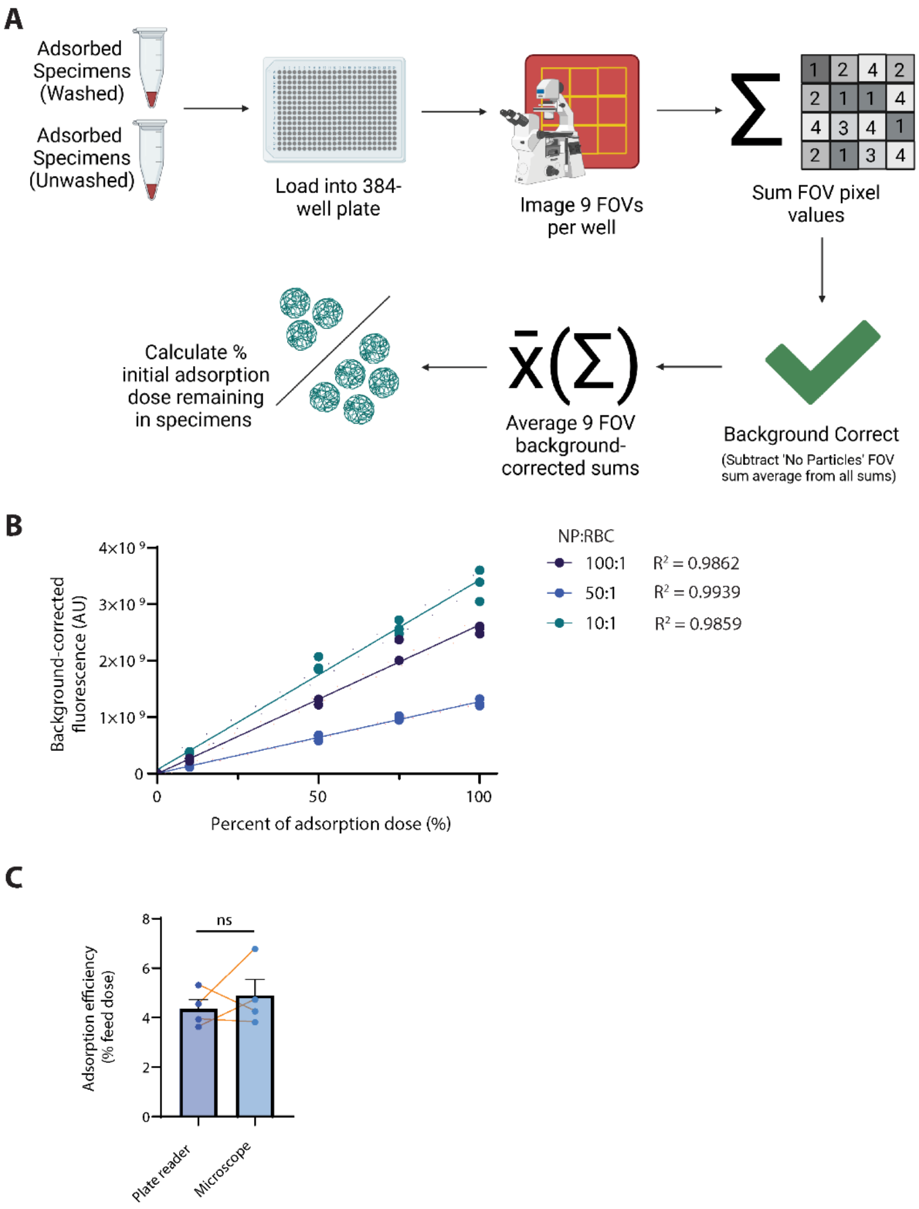

Fig. S7.

Adsorption efficiency assay description. (A) Schematic showing the workflow of determining "adsorption efficiency" as a fraction of remaining fluorescent cargo signal following post-adsorption RBC-nanoparticle complex washes over total fluorescent cargo signal detected in adsorption feed dose prior to removal of non-adsorbed nanoparticles. (B) Linearity determinations of assay at 3 different nanoparticle adsorption feed dose ratios. Two exposure settings were utilized, one for 100:1 and 50:1 and another for 10:1. Linear regression along with dotted 95% confidence intervals are displayed. n = 3. (C) Correlation of adsorption efficiency measures between microscopy fluorimetry measures as described in fig. S7A and standard plate reader fluorimetry measures. n = 4. Error bars indicate standard error of the mean. Orange lines connecting data points indicate the same specimen. ns = not significant; RBC = red blood cell, FOV = field of view, NP = nanoparticle, AU = arbitrary units.

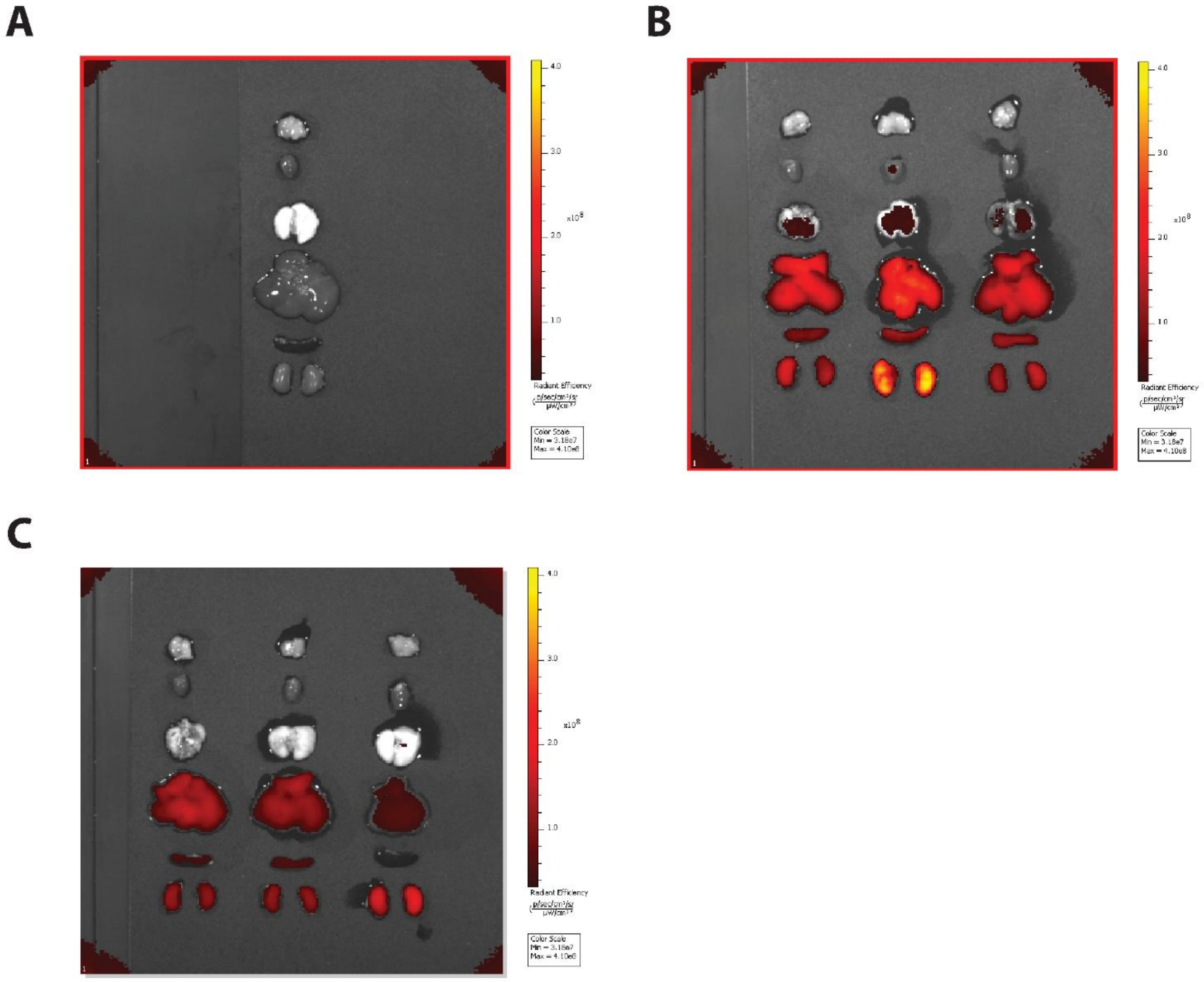

Fig. S8.

Full IVIS images displaying radiant efficiency [(photons/s/cm^2/sr)/(μW/cm^2)] from *in vivo* biodistribution experiments of PACE60 nanoparticles encapsulating Cy5-ssDNA. Organs of mice from the following groups: (A) no-nanoparticle control, (B) hitchhiking group, (C) free nanoparticles at 12% adsorption feed dose group. Experimental n = 3, control n = 1. Color scale: minimum = 3.18 x 10^7, maximum = 4.10 x 10^8. Organs shown top to bottom include: thymus, heart, lungs, liver, spleen, kidneys. Representative images of each group are duplicated in main text Figure 6A. IVIS = in vivo imaging system, PACE60 = poly(amine-co-ester) synthesized with 60 mol% 15-pentadecanolide, ssDNA = single-stranded DNA.

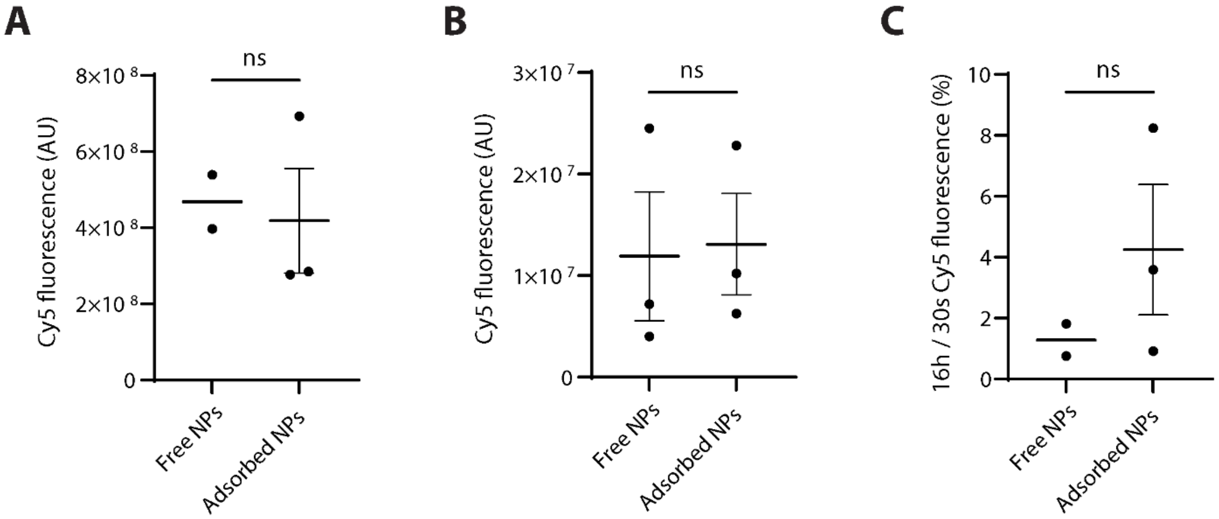

Fig. S9.

Whole blood fluorimetry measurements examining PACE60 nanoparticle pharmacokinetics between hitchhiked and free nanoparticle groups. (A) Blood nanoparticle cargo signal 30 s post-administration. (B) Blood nanoparticle cargo signal 16 h post-administration at time of organ procurement. (C) Fraction of nanoparticle cargo signal detected at 16 h over signal detected at 30 s post-administration, reflective of nanoparticle cargo signal remaining in circulation at time of organ procurement. n = 2-3; ns = not significant. Lines and error bars indicate mean and standard error of the mean, respectively. PACE60 = poly(amine-co-ester) synthesized with 60 mol% 15-pentadecanolide, NP = nanoparticles, h = hours, AU = arbitrary units.

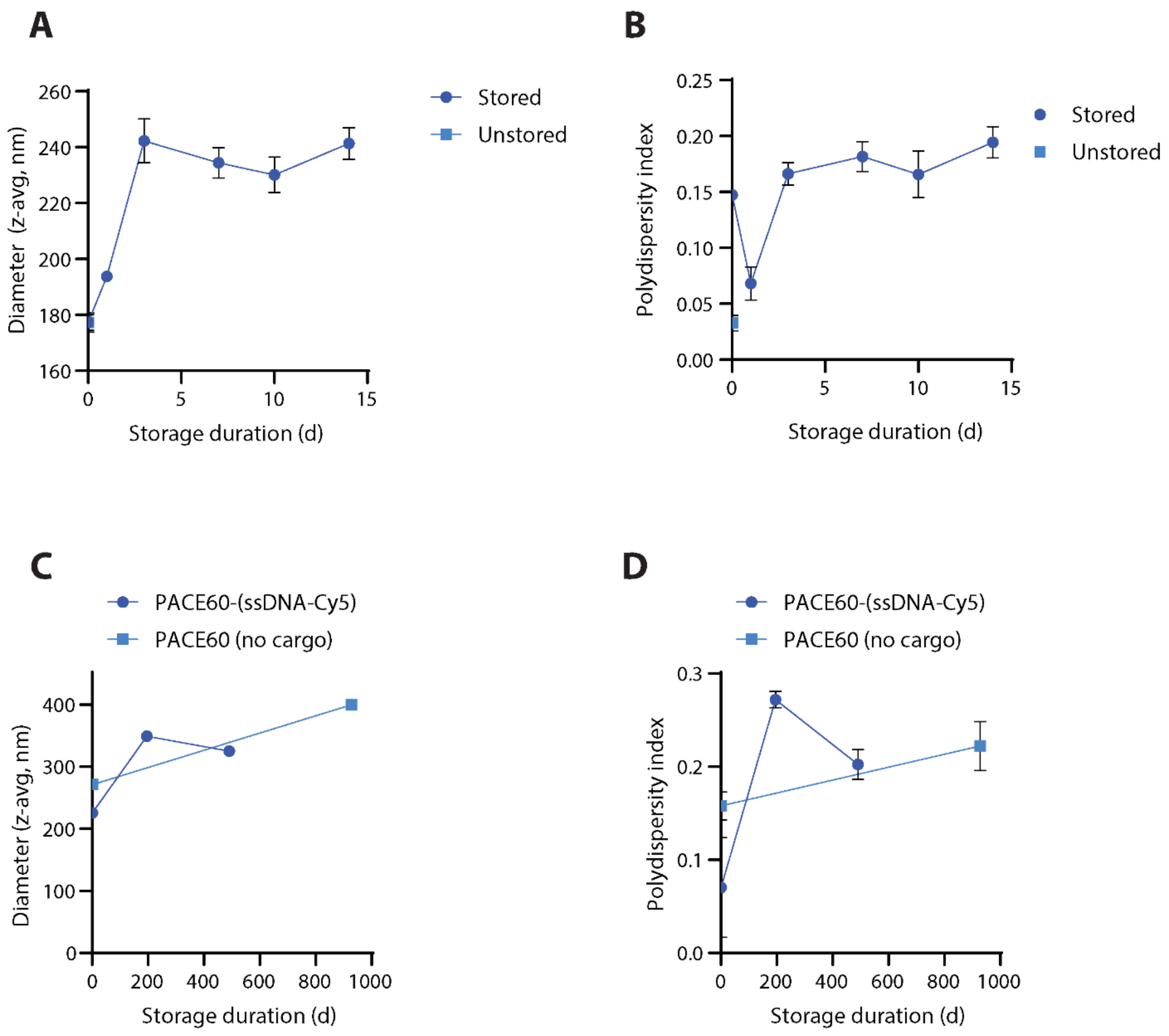

Fig. S10.

Storage stability of PACE60 nanoparticles. (A) Diameter (z-avg) and (B) polydispersity index of PACE60 nanoparticles as a function of storage at -80°C when flash frozen in aqueous media. n = 3 (batch replicates). Symbols and error bars indicate mean and standard error of the mean, respectively. "Unstored" data at $x = 0$ (light blue squares) correspond to measurements taken prior to freezing. (C) Diameter (z-avg) and (D) polydispersity index of PACE60 nanoparticles as a function of storage at -80°C when flash frozen in aqueous media. n = 3 (measurement replicates). Symbols and error bars indicate mean and standard error of the mean, respectively. Data at x = 0 corresponds to data taken prior to freezing. PACE60 = poly(amine-co-ester) synthesized with 60 mol% 15-pentadecanolide, ssDNA = single-stranded DNA, d = days.

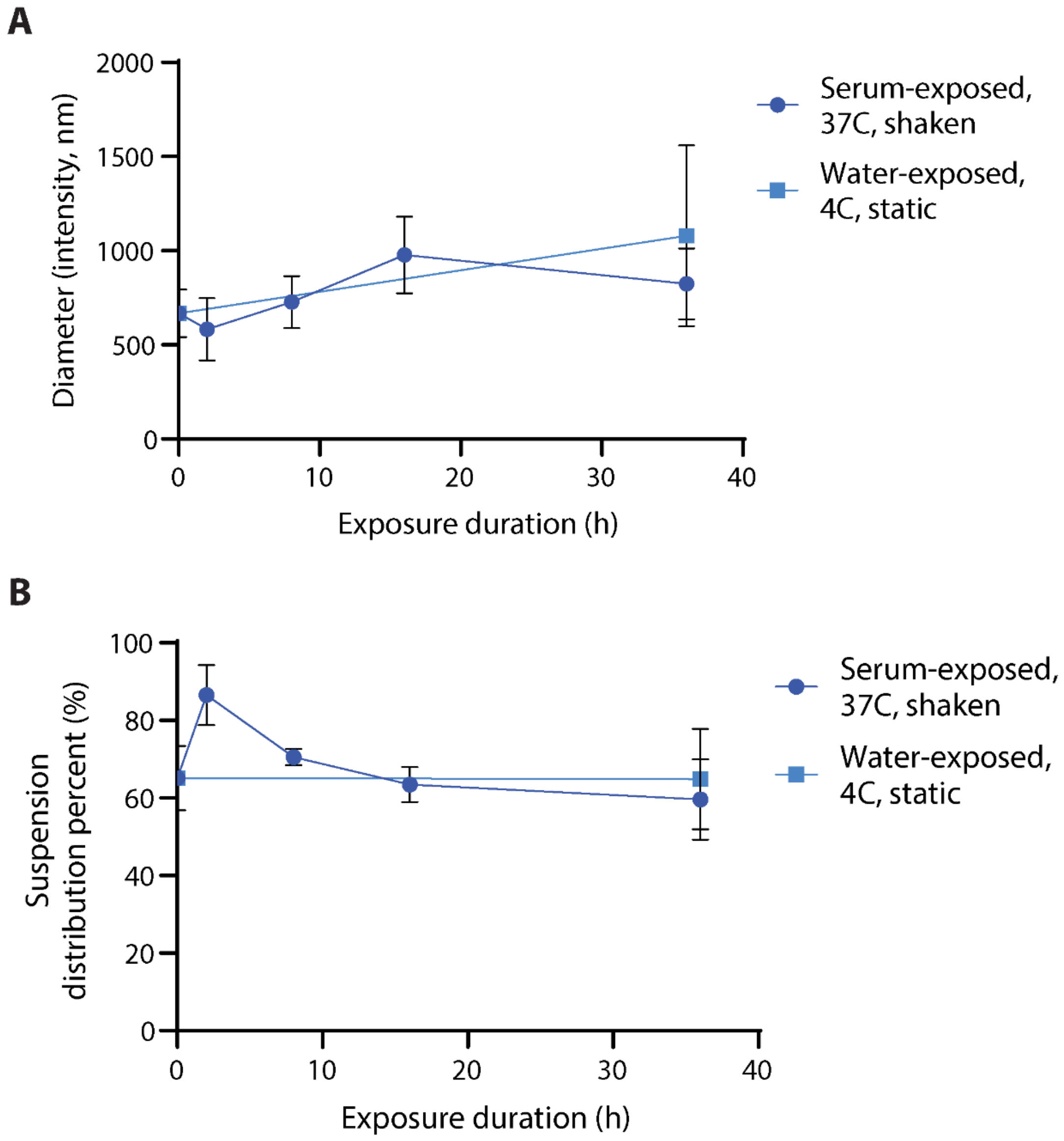

Fig. S11.

Serum stability of PACE60 nanoparticles. (A) Diameter (intensity) of PACE60 nanoparticles exposed to 100% mouse serum for various time intervals then measured via DLS in 10% mouse serum + 90% water. As measurements were confounded by the presence of plasma proteins, the majority peak on the size distribution was assumed to be the nanoparticle peak. The percentage of this peak out of the total size distribution is reported in (B). Nanoparticles stored in their stock solution in water at 4°C for the study period were also measured as a control. n = 3 (measurement replicates). Symbols and error bars indicate mean and standard deviation, respectively. PACE60 = poly(amine-co-ester) synthesized with 60 mol% 15-pentadecanolide, DLS = dynamic light scattering, h = hours.

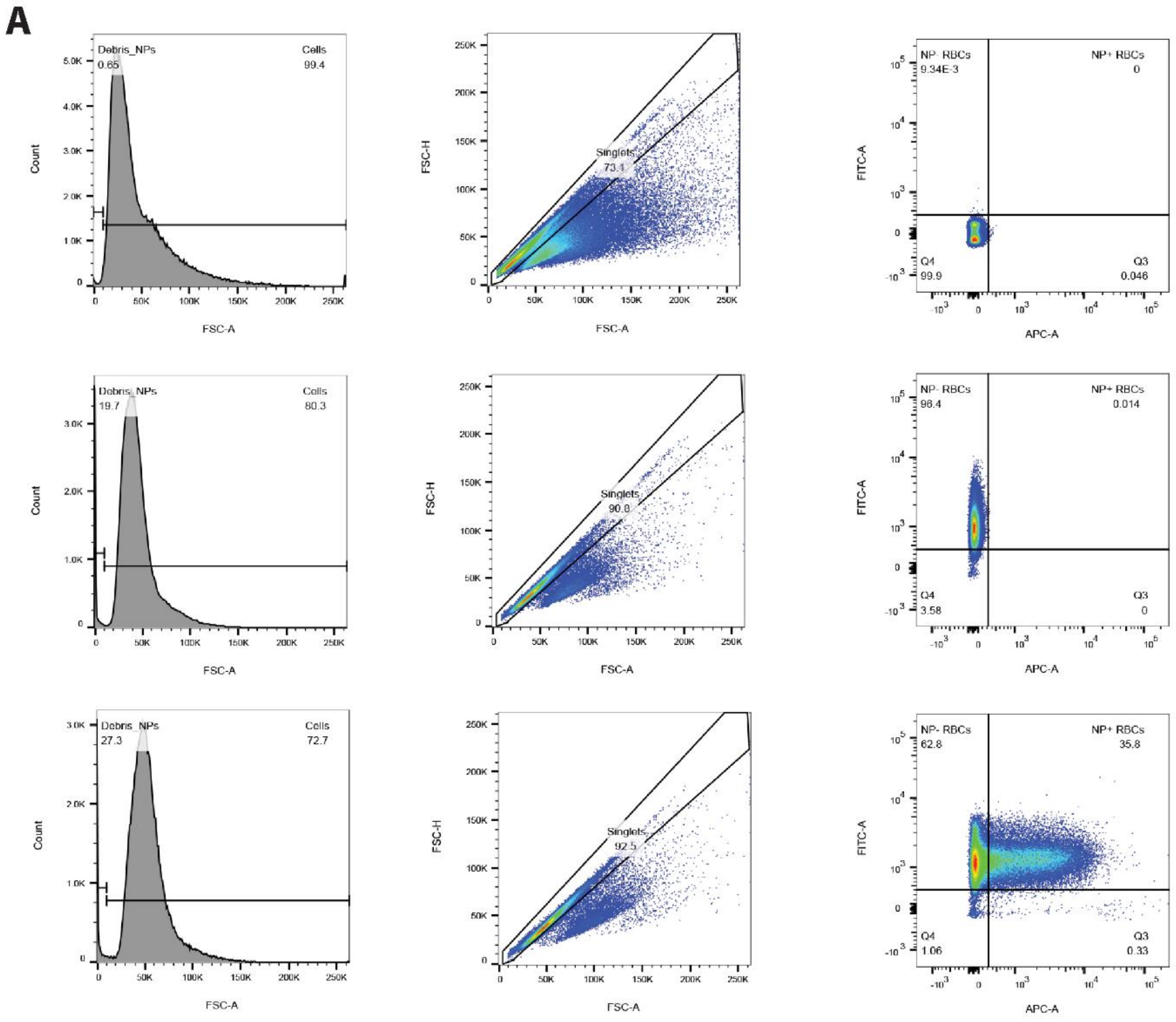

Fig. S12.

Flow cytometry gating strategy on the BD LSRII instrument. (A) Cells were gated on forward scatter (FSC-A) to exclude debris, followed by doublet-exclusion (FSC-H vs FSC-A), and finally FITC positivity (anti-RBC TER-119 antibody clone conjugate) and Cy5 (nanoparticle cargo, APC channel) positivity. Top row represents no antibody, no nanoparticle control. Middle row represents antibody-labeled, no nanoparticle control. Bottom row represents a specimen labeled with the anti-RBC antibody and with adsorbed nanoparticles. FSC = forward scatter, RBC = red blood cell.

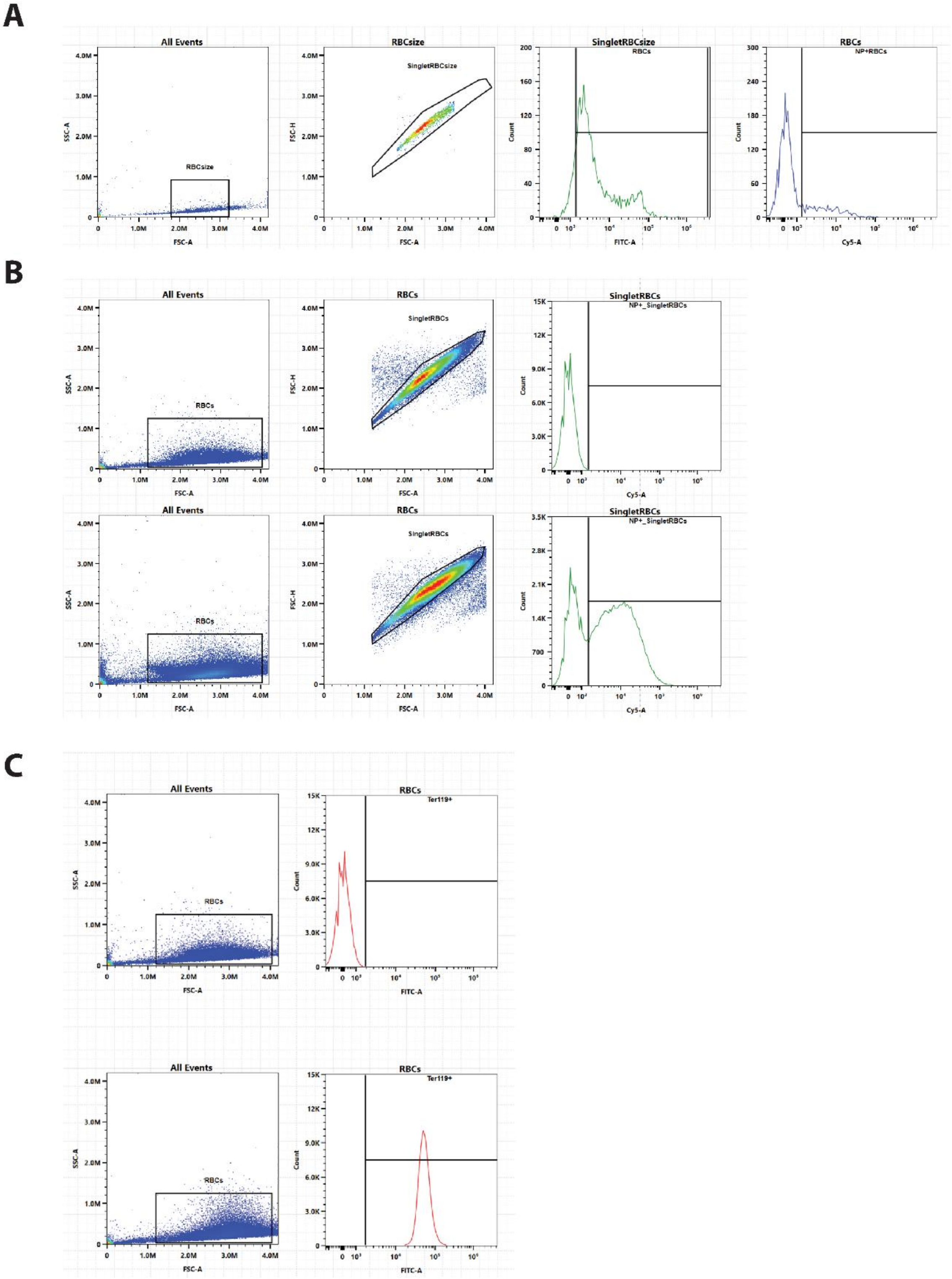

Fig. S13.

Flow cytometry gating strategy on the Cytek Aurora spectral flow cytometer. (A) Cells were gated according to forward scatter (FSC-A) to obtain uniformly sized events, followed by

doublet-exclusion (FSC-H vs FSC-A), anti-RBC FITC positivity (FITC-A), and finally Cy5 nanoparticle cargo positivity (Cy5-A). (B) For experiments examining glutaraldehyde- and trypsin-treated RBCs, only the size and doublet exclusion gates were utilized prior to determination of Cy5 positivity. Top row represents a no-nanoparticle control. Bottom row represents a specimen with adsorbed nanoparticles. (C) For experiments examining glutaraldehyde- and trypsin-treated RBCs, the initial size gate utilized was correlated to anti-erythroid labeling, corresponding to 100% positivity. For all flow cytometry carried out on the Cytek Aurora, autofluorescence extraction was utilized. Separate unstained controls were utilized for glutaraldehyde-fixed RBCs given differential autofluorescence profiles observed. FSC = forward scatter, RBC = red blood cell.

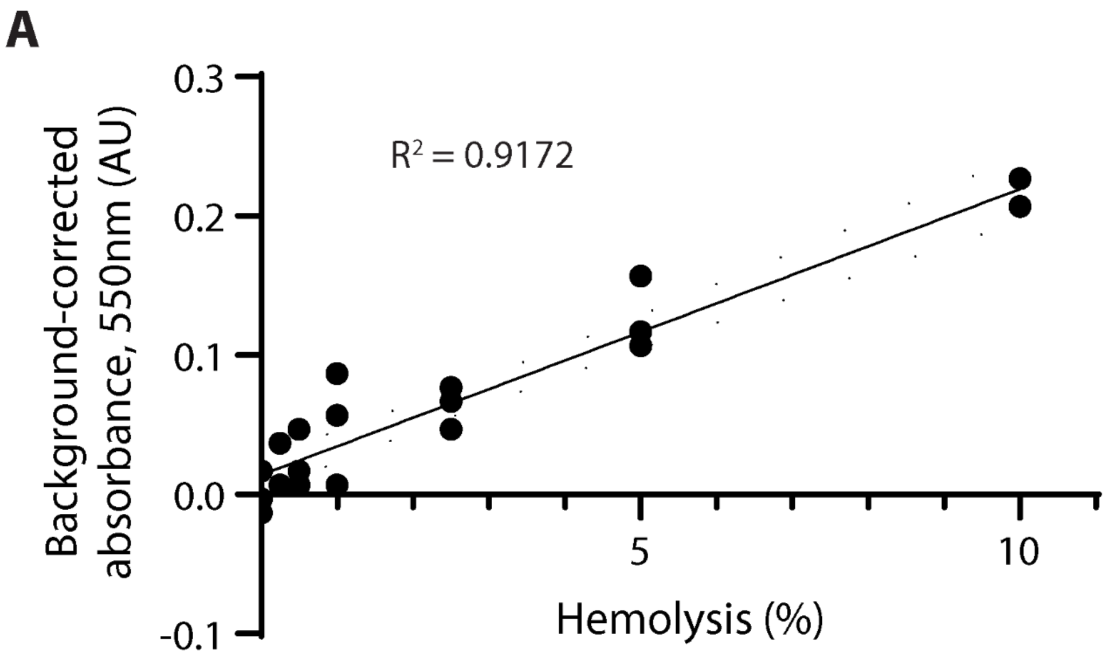

Fig. S14.

Hemolysis assay linearity. (A) Hemolysis assay linearity confirmation. Linear regression along with dotted 95% confidence intervals are displayed. n = 3. AU = arbitrary units.

Table S1.

Characterization of nanoparticle formulations utilized throughout this study. "/3Cy5Sp/" indicates a 3' Cy5 conjugation. PACE60 = poly(amine-co-ester) synthesized with 60 mol% 15-pentadecanolide, PLGA (50:50) = poly(lactic-co-glycolic) acid synthesized with a 1:1 monomer ratio, [C] = concentration, NA = not available, PDI = polydispersity index.

Polymer	Cargo	Cargo [C]	DNA Sequence (5' - 3')	Size (z-avg, nm)	PDI	Zeta (mV)
PACE60	Cy5-ssDNA	44 nmol	GGC TAA GCT TGT ACA ATA A /3Cy5Sp/	247.7	0.096	25.0
PACE60	Cy5-dsDNA	50 nmol	TAG TGG TCC ATA TAG AGG TAC /3Cy5Sp/ + complement without Cy5	260.0	0.102	3.2
PACE60	Cy5-ssDNA	50 nmol	GTA CCT CTA TAT GGA CCA CTA /3Cy5Sp/	225.7	0.070	23.0
PACE60	Cy5-ssDNA	50 nmol	GTA CCT CTA TAT GGA CCA CTA /3Cy5Sp/	282.9	0.110	17.9
PACE60	Cy5-ssDNA	50 nmol	GTA CCT CTA TAT GGA CCA CTA /3Cy5Sp/	203.7	0.084	18.9
PACE60	DiD	0.5% by mass	NA	250.5	0.058	27.5
PACE60	None	NA	NA	271.1	0.158	23.4
PACE60	DiI	0.5% by mass	NA	177.4	0.147	NA
Polystyrene	None	NA	NA	206.4	0.020	-42.9
PLGA (50:50)	None	NA	NA	229.8	0.085	-26.1

Table S2.

Materials utilized throughout this study.

Material	Manufacturer	Product Number / Sequence
15-Pentadecanolide; >98%	Sigma-Aldrich	W284009
anti-(mouse erythroid cells)-FITC; clone TER-119	Biolegend	116206 (RRID: AB_313706)
Chloroform; HPLC grade	Sigma-Aldrich	366927
CPDA-1	Jorgensen Laboratories	J-520Q
Dichloromethane	Sigma-Aldrich<br>Millipore Sigma	270997<br>75-09-2
DiD	Biotium	60014-1mg
Diethyl sebacate, 98%	Sigma-Aldrich	246077
Dimethyl sulfoxide; ACS reagent grade	JTBaker	9224-01
Diphenyl ether; 99%	Sigma-Aldrich	P24101
dsDNA-Cy5 oligonucleotide	IDT	TAG TGG TCC ATA TAG AGG TAC /3Cy5Sp/ + complementary strand without Cy5
Dulbecco's phosphate buffered saline; - CaCl2, - MgCl2	gibco	14190-144
EDTA; disodium salt, dihydrate, crystal	JTBaker	8993-01
FITC rat IgG2b/k isotype conjugate	Biolegend	400605 (RRID: AB_326549)
Gluteraldehyde solution; grade I, 8% in H2O	Thomas Scientific	C988U62
Heparin sodium salt from porcine intestinal mucosa; grade I-A, BioReagent	Sigma-Aldrich	H3149
Hexane; HPLC grade, >97%	Sigma-Aldrich	34859
Neuraminidase; Type V, (*Clostridium welchii*)	Sigma-Aldrich	N2876
N-methyldiethanolamine; >99%	Sigma-Aldrich	471828
Normal mouse serum	Invitrogen	10410
Novozym 435	Sigma-Aldrich	L4777
Phosphate buffered saline; pH 7.4, - CaCl2, - MgCl2	gibco	10010-023
Poly(D,L-lactide-co-glycolide), 50:50	Sigma-Aldrich	26780-50-7
Poly(vinyl alcohol)	Sigma-Aldrich<br>Sigma-Aldrich	P8136<br>9002-89-5

Polystyrene microparticles; 0.2 um	Supelco	95581
ssDNA-Cy5 oligonucleotide 1	IDT	GGC TAA GCT TGT ACA ATA A /3Cy5Sp/
ssDNA-Cy5 oligonucleotide 2	Keck Biotechnology Resource Laboratory, Yale University	GTA CCT CTA TAT GGA CCA CTA /3Cy5Sp/
Sterile Acrodisc™ WBC syringe filter with Leukosorb membrane; 25 mm	Pall Corporation	AP-4951
Tris base; molecular biology grade, ultra pure	dot scientific inc.	DST60040
Trypsin-Ethylenediaminetetraacetic acid disodium salt dihydrate; 0.25%, phenol red	Gibco	25200056
UltraPure distilled water; DNAse, RNAse Free	Invitrogen	10977-015
Vybrant DiO cell-labeling solution	Invitrogen	V22886

Movie S1.

Confocal z-stack montage of isolated red blood cells (red) with membrane-adsorbed PACE60 nanoparticles (cyan) from the field-of-view reported in Figure 1A; scale bar = 2 µm. Brightness/contrast of RBC and nanoparticle channels independently adjusted to facilitate visualization of spatial relationship. PACE60 = poly-amine-co-ester synthesized with 60 mol% 15-pentadecanolide, RBC = red blood cell.

Data S1.

Numerical data (CSV) reported in main text and supplementary materials figures. For figures displaying relative measurements, original data are provided.
